# Mendelian randomization analysis suggests no associations of human herpes viruses with amyotrophic lateral sclerosis

**DOI:** 10.3389/fnins.2023.1299122

**Published:** 2023-12-12

**Authors:** Qingcong Zheng, Du Wang, Rongjie Lin, Yuchao Chen, Haoen Huang, Zixing Xu, Chunfu Zheng, Weihong Xu

**Affiliations:** ^1^Department of Spinal Surgery, the First Affiliated Hospital of Fujian Medical University, Fuzhou, China; ^2^Department of Orthopedics, National Regional Medical Center, Binhai Campus of the First Affiliated Hospital, Fujian Medical University, Fuzhou, China; ^3^Arthritis Clinical and Research Center, Peking University People's Hospital, Beijing, China; ^4^Department of Orthopedic Surgery, Fujian Medical University Union Hospital, Fuzhou, China; ^5^Department of Paediatrics, Fujian Provincial Hospital South Branch, Fuzhou, China; ^6^Department of Microbiology, Immunology and Infectious Diseases, University of Calgary, Calgary, AB, Canada

**Keywords:** amyotrophic lateral sclerosis, human herpes virus, Mendelian randomization, causal association, neurodegenerative diseases

## Abstract

**Background:**

The causal associations between infections with human herpes viruses (HHVs) and amyotrophic lateral sclerosis (ALS) has been disputed. This study investigated the causal associations between herpes simplex virus (HSV), varicella-zoster virus (VZV), Epstein–Barr virus (EBV), cytomegalovirus (CMV), HHV-6, and HHV-7 infections and ALS through a bidirectional Mendelian randomization (MR) method.

**Methods:**

The genome-wide association studies (GWAS) database were analyzed by inverse variance weighted (IVW), MR-Egger, weighted median, simple mode, and weighted mode methods. MR-Egger intercept test, MR-PRESSO test, Cochran’s Q test, funnel plots, and leaveone-out analysis were used to verify the validity and robustness of the MR results.

**Results:**

In the forward MR analysis of the IVW, genetically predicted HSV infections [odds ratio (OR) = 0.9917; 95% confidence interval (CI): 0.9685–1.0154; *p* = 0.4886], HSV keratitis and keratoconjunctivitis (OR = 0.9897; 95% CI: 0.9739–1.0059; *p* = 0.2107), anogenital HSV infection (OR = 1.0062; 95% CI: 0.9826–1.0304; *p* = 0.6081), VZV IgG (OR = 1.0003; 95% CI: 0.9849–1.0160; *p* = 0.9659), EBV IgG (OR = 0.9509; 95% CI: 0.8879–1.0183; *p* = 0.1497), CMV (OR = 0.9481; 95% CI: 0.8680–1.0357; *p* = 0.2374), HHV-6 IgG (OR = 0.9884; 95% CI: 0.9486–1.0298; *p* = 0.5765) and HHV-7 IgG (OR = 0.9991; 95% CI: 0.9693–1.0299; *p* = 0.9557) were not causally associated with ALS. The reverse MR analysis of the IVW revealed comparable findings, indicating no link between HHVs infections and ALS. The reliability and validity of the findings were verified by the sensitivity analysis.

**Conclusion:**

According to the MR study, there is no evidence of causal associations between genetically predicted HHVs (HSV, VZV, EBV, CMV, HHV-6, and HHV-7) and ALS.

## 1 Introduction

Amyotrophic lateral sclerosis is a rapidly progressive and fatal neuronal disease characterized by progressive degeneration of motor neurons in the brain and spinal cord, ultimately leading to almost total skeletal muscle paralysis (van Es et al., 2017). Patients with ALS often present with progressive weakness and atrophy of the extremities, gradually leading to an inability to walk, talk, swallow, and breathe (Taylor et al., 2016). ALS is a rare disease with an incidence of 3.1 per 100,000 person-years (Vasta et al., 2022), with familial ALS (fALS) accounting for 10% of cases and sporadic ALS (sALS) accounting for 90% of cases (Pham et al., 2020) and the average survival after diagnosis is in the range of 3–5 years (Brown and Al-Chalabi, 2017). The number of ALS cases is expected to increase by 69% from 2015 to 2040 due to global aging (Arthur et al., 2016). However, little is known despite the time and money spent investigating ALS’s pathogenic mechanisms. ALS is believed to be caused by genetic and environmental interactions (Celeste and Miller, 2018; Schram et al., 2020; Julian et al., 2021; Motataianu et al., 2022; Goutman et al., 2023), with some investigators suggesting that viruses are an important environmental factor in ALS (Celeste and Miller, 2018; Castanedo-Vazquez et al., 2019).

Human herpes viruses are one of the largest families of double-stranded DNA viruses, comprising three main subfamilies: α, β, and γ-herpesviruses. α-herpesviruses include HSV-1, HSV-2, and VZV (HSV-3), β-herpesviruses include CMV (HHV-5), HHV-6, and HHV-7, and γ-herpesviruses include EBV (HHV-4) and HHV-8 (Sehrawat et al., 2018). Epidemiological data showed that billions of people were infected with HSV-1, and 500 million had HSV-1/HSV-2 genital infections in 2016 (James et al., 2020). A global disease burden on VZV reported that new cases surpassed 80 million in 2019 alone and continue to rise (Huang et al., 2022). More than 90% of adults worldwide are chronically infected with EBV (Huang et al., 2021). Moreover, the general population has a seropositivity rate of 83% for CMV IgG antibodies (Zuhair et al., 2019). Approximately 80–90% of adults worldwide have herpesviruses (Khalesi et al., 2023).

Studies have shown that enteroviruses and herpesviruses are the two most common viruses infecting hospitalized patients’ central nervous system (CNS) (Roshdy et al., 2023). The α/β/γ-herpesviruses are neurotoxic and neurotropic viruses and are often considered important risk factors for neurodegenerative disease (NDD) (Osorio et al., 2022). However, the relationship between HHVs and ALS is unknown. A study reported that herpesvirus infection and ALS can flare up simultaneously. Nevertheless, there is no way to determine if it is accidental or intentional (Ferri-de-Barros and Moreira, 2010). Some investigators have suggested that HSV-1 can latently infect the trigeminal ganglion and may be a causative factor in ALS (Feng et al., 2022). In addition, the pathogenic risk of ALS was slightly associated with HHV-6 seropositivity in a case–control study (Cermelli et al., 2003). In contrast, no significant correlation was found between ALS and IgG antibodies to HSV and CMV in an immunological evaluation of early ALS (Provinciali et al., 1988). These studies with conflicting conclusions may be due to methodological flaws, including confounders and reverse causality in observational studies. We have no method of determining the association, let alone the causality, between HHVs and ALS.

Random control trials (RCTs) are a type of experimental research methodology that evaluates the effect of a causative factor or a treatment regimen on a disease by randomizing study subjects into groups, implementing different interventions for different groups, and finally comparing the results. RCTs are the gold standard for clinical diagnosis and can determine the causal association between exposure and outcome. There are no reported RCTs on the association between HHVs and ALS, mainly due to the strict constraints of the design process and medical ethics and few reports of observational studies and their mixed conclusions, which makes it important to carry out MR analyses in this context. MR is an epidemiological investigation method based on instrumental variables (IVs) to analyze summary-level data from GWAS, which can greatly reduce confounding bias and consistently and reliably infer causality between exposures and outcomes due to the stochastic nature of the genetic variants and the fact that alleles are not affected by disease (Emdin et al., 2017). Two-sample Mendelian randomization (TSMR) refers to using genetic variants as IVs in both exposure and outcome samples to investigate the effects of modifiable risk factors for disease. Although lower than RCTs, the strength of evidence from MR analyses is stronger than observational studies (Davies et al., 2018). Particularly in rare diseases, MR can achieve more reliable results by analyzing and evaluating much larger sample sizes than in conventional clinical trials. Therefore, our study investigated whether there is a causal association between HHVs and ALS using a bidirectional TSMR method based on GWAS data.

## 2 Materials and methods

### 2.1 Study design

This study strengthened epidemiological observational studies using Mendelian randomization (STROBE-MR) (Skrivankova et al., 2021). All data were obtained from the publicly available GWAS database without re-ethical approval. In MR, SNPs as IVs must fulfill the following three assumptions. (1) The relevance assumption: IVs are closely related to exposure; (2) the independence assumption: the IVs are not associated with the potential confounders; (3) the exclusion restriction assumption: IVs affect outcomes only through the exposure pathway (no directional pleiotropy) (Supplementary Figure S1).

### 2.2 GWAS data sources

The summary-level statistics for all cases and controls in this study are of European ancestry, and the study subjects were residents recruited from multiple research centers in Europe to minimize bias due to race-related confounding factors. GWAS data for HHV-8 of suitable European ancestry could not be found and were therefore not analyzed.

#### 2.2.1 Exposure

The exposure factors and the dataset for this study were as follows. HSV: the finn-b-AB1_HERPES_SIMPLEX (1,595 cases, 211,856 controls and 16,380,457 SNPs), finn-b-H7_HERPESKERATITIS (573 cases, 209,287 controls and 16,380,429 SNPs) and finn-b-AB1_ANOGENITAL_HERPES_SIMPLEX (10,118,743 SNPs) datasets were searched in the latest FinnGen.^1^ VZV IgG: The GCST90006928 (25,472,218 SNPs) dataset was searched in GWAS.^2^ EBV: the finn-b-AB1_EBV (1,238 cases, 213,666 controls, and 16,380,461 SNPs) dataset was searched for in FinnGen (r9). CMV IgG: The ieu-b-4900 (7,002,835 SNPs) dataset was searched in the Integrative Epidemiology Unit (IEU, https://gwas.mrcieu.ac.uk/). HHV-6 IgG: The GCST90006902 (25,472,218 SNPs) dataset was searched in the GWAS database. HHV-7 IgG: The GCST900069028 (25,472,218 SNPs) dataset was searched in the GWAS database.

#### 2.2.2 Outcome

The ALS dataset for GCST90027164 (27,205 cases, 110,881 controls, and 10,461,755 SNPs) was searched in the GWAS database. After cleaning and conversion, we saved the data downloaded from the FinnGen database in GWAS database format. After comparing the sources of participants in the eight datasets for HHVs with the one dataset for ALS, we consider the samples for HHVs and ALS to be independent. We list these data in Supplementary Table S1.

### 2.3 Selection of instrumental variables

A critical step in MR analysis is to obtain valid IVs. We extracted SNPs (*p* < 5 × 10^−5^) with significant correlation with HSV, VZV, EBV, CMV, HHV-6, and HHV-7 from the eight exposure datasets. Subsequently, we performed linkage disequilibrium (LD) analysis on the obtained SNPs with “*r* ^2^ < 0.001, 10,000 = kb” by using the “clump_data” function to exclude the mutual linkage SNPs and to discard non-biallelic SNPs. We used the F-statistic to assess the strength of the association between the selected IVs and exposure. F-statistic is calculated as F = (β/se(β)) ^2^ (Zhang et al., 2023) when *F* > 10 indicates that IVs are strong instrumental variables, which avoids the bias caused by weak IVs. In addition, the summary set may produce errors if the effect alleles for the SNP effects are different in the GWAS data for exposure and outcome. Therefore, we used the “harmonise_data” function to test the causal direction of the screened SNPs in exposure and outcome, excluded palindromic alleles, and selected the SNPs with “TRUE” results for MR analysis.

### 2.4 TSMR analysis

The data in this study were analyzed based on the “TwoSampleMR” package of R version 4.2.3 software. Analyses included Forward MR with HHVs infection or IgG as the exposure and ALS as the outcome and reverse MR with ALS as the exposure and HHVs infection or IgG as the outcome. We chose MR Egger, weighted median, IVW, simple mode, and weighted mode methods to calculate the causal relationship between exposure and outcome, and IVW was the most valid and reliable of these methods. We then perform sensitivity analyses. Cochran’s Q-statistic was used to test for heterogeneity (*p* < 0.05) between SNPs in MR-Egger and IVW analyses to assess the robustness of IVs (Bowden et al., 2019). Heterogeneity was additionally visualized by constructing a funnel plot of the IVs. MR-PRESSO can detect outliers that may bias the results and give a causal change in exposure and outcome after removing the outlier (Verbanck et al., 2018). Therefore, when MR-PRESSO detects an outlier, we exclude the SNPs and re-perform the MR analysis and evaluation. Pleiotropy refers to the fact that some IVs affect outcomes through pathways other than exposure, which would seriously affect the reliability of the causal association between exposure and outcome (Davey Smith and Hemani, 2014). We used the MR-Egger intercept for bias detection and effect estimation. When the “MR_pleiotropy_test” function calculates *p* < 0.05, it means that there is directional pleiotropy (Bowden et al., 2015). Leave-one-out analysis estimates the effect of the remaining SNPs on the outcome by sequentially removing individual SNPs and then performing the IVW analysis again, which can determine whether any single SNPs drive causality.

## 3 Results

### 3.1 Screening of instrumental variables

In forward MR, we ended up with 69, 55, 56, 65, 6, 7, 5, and 6 SNPs that were closely associated with HSV infections, HSV keratitis and keratoconjunctivitis, anogenital HSV infection, VZV IgG, EBV, CMV IgG, HHV-6 IgG, and HHV-7 IgG, respectively. In reverse MR, 9, 9, 8, 10, 9, 8, 10, and 9 SNPs were obtained when ALS was used as the exposure corresponding to HSV infections, HSV keratitis and keratoconjunctivitis, anogenital HSV infection, VZV IgG, EBV, CMV IgG, HHV-6 IgG, HHV-7 IgG, respectively. These SNPs were all strong instrumental variables (F-statistic >10). A total of sixteen MR analyses were performed in this study, and details of the screened IVs can be found in Supplementary Tables S2–S17.

### 3.2 Forward MR

**Figure 1:**
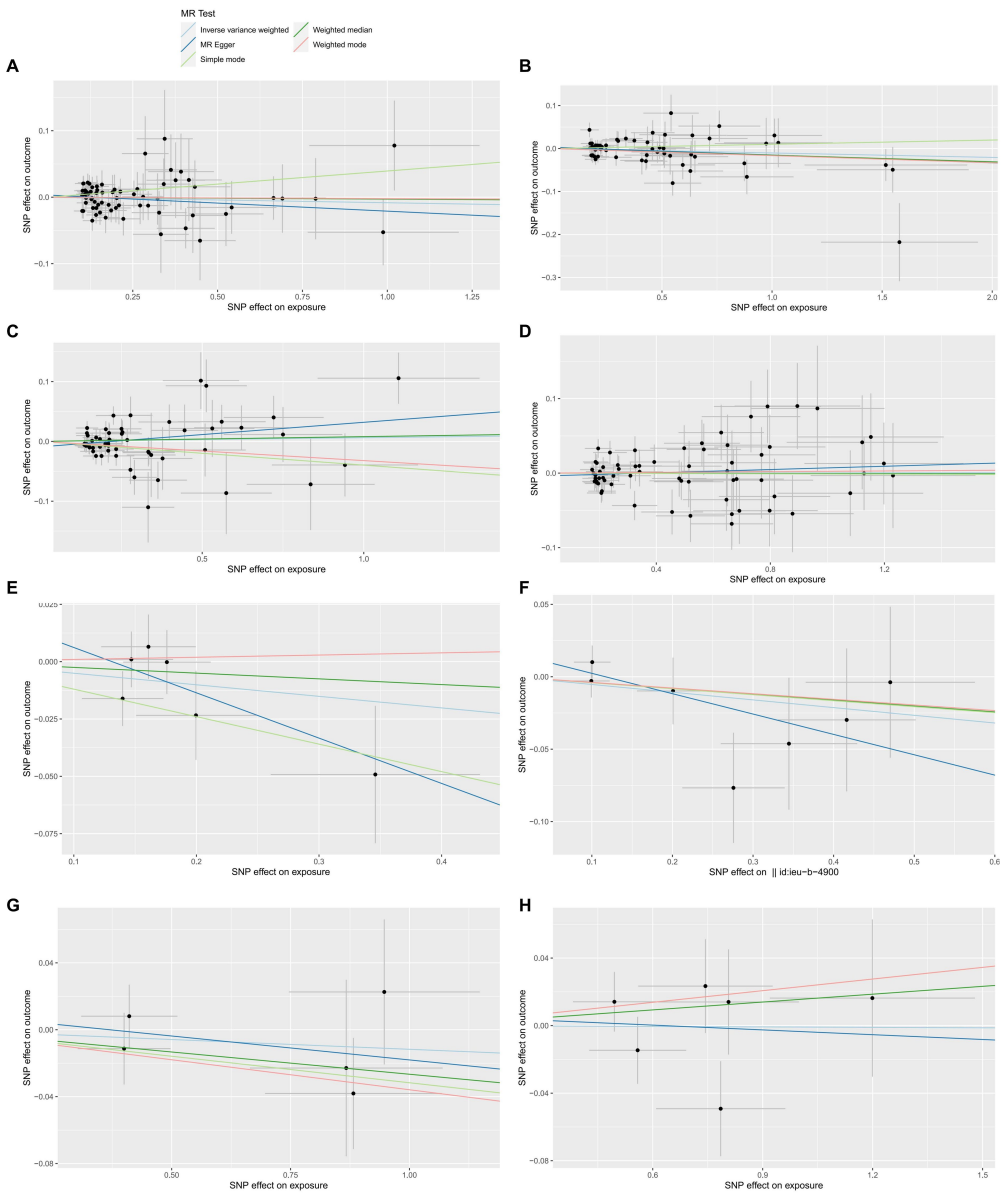
The forward MR effect of HHVs and ALS. Scatter plots for MR-Egger, weighted median, IVW, simple mode and weighted mode methods highlighting the effect of HSV infections **(A)**, HSV keratitis and keratoconjunctivitis **(B)**, anogenital HSV infection **(C)**, VZV IgG **(D)**, EBV **(E)**, CMV IgG **(F)**, HHV-6 IgG **(G)**, HHV-7 IgG **(H)**, on ALS.

**Figure 2:**
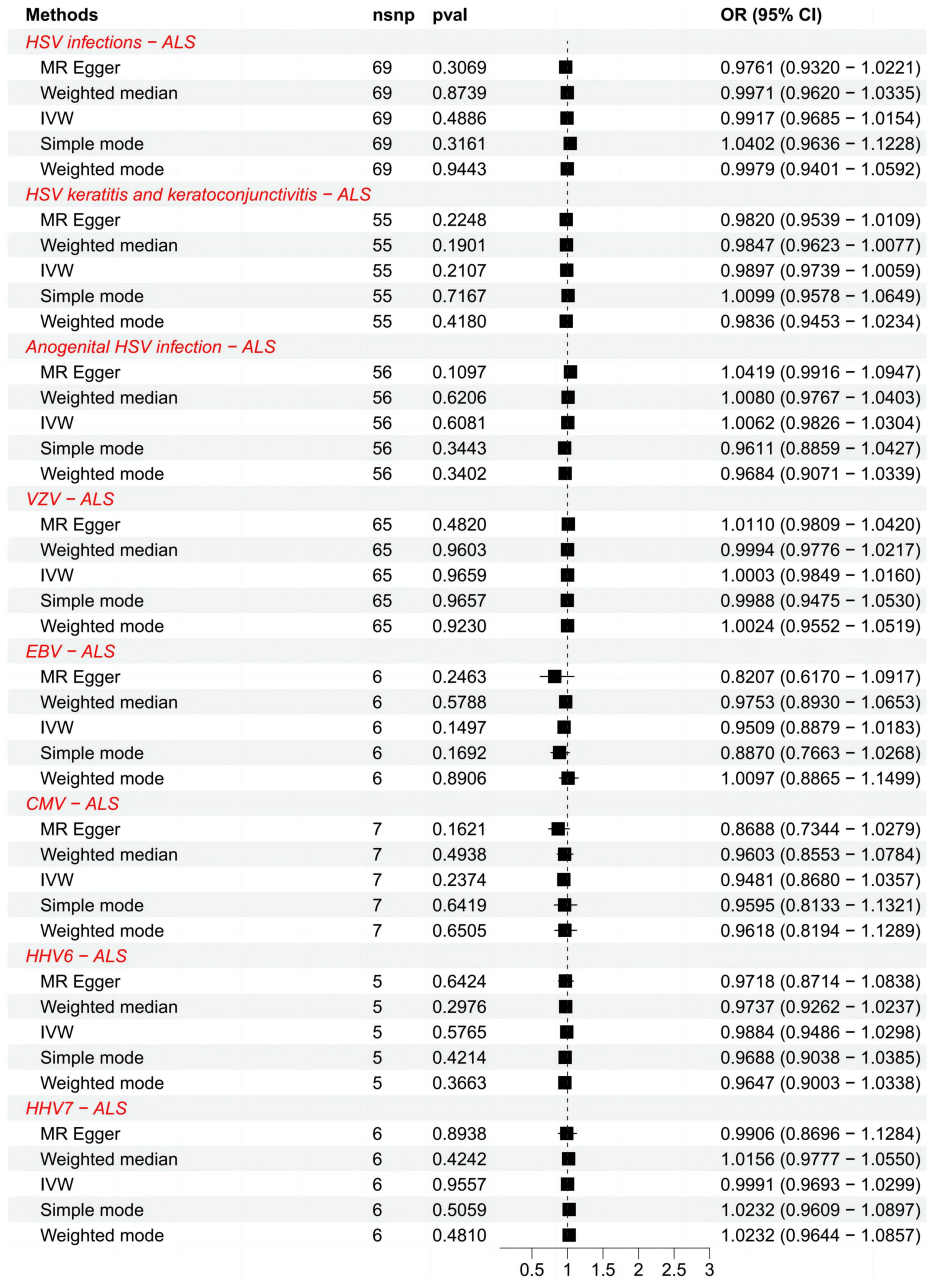
Forest plots of causal effect estimates in the forward MR analysis. SNP, single-nucleotide polymorphism; ALS, amyotrophic lateral sclerosis; IVW, inverse variance weighted; OR, odds ratio; 95% CI, 95% confidence interval.

We list the TSMR and the sensitivity analysis results of HHVs and ALS in Table 1. Using IVW as the primary method, it can be seen that genetically predicted HSV infections (OR = 0.9917; 95% CI: 0.9685–1.0154; *p* = 0.4886), HSV keratitis and keratoconjunctivitis (OR = 0.9897; 95% CI: 0.9739–1.0059; *p* = 0.2107), anogenital HSV infection (OR = 1.0062; 95% CI: 0.9826–1.0304; *p* = 0.6081), VZV IgG (OR = 1.0003; 95% CI: 0.9849–1.0160; *p* = 0.9659), EBV IgG (OR = 0.9509; 95% CI: 0.8879–1.0183; *p* = 0.1497), CMV (OR = 0.9481; 95% CI: 0.8680–1.0357; *p* = 0.2374), HHV-6 IgG (OR = 0.9884; 95% CI: 0.9486–1.0298; *p* = 0.5765) and HHV-7 IgG (OR = 0.9991; 95% CI: 0.9693–1.0299; *p* = 0.9557) were not causally associated with ALS, which completely agrees with the conclusions reached by the four methods: MR-Egger, weighted median, simple mode, and weighted mode (Figure 1). Notably, 57 SNPs obtained from the dataset associated with anogenital HSV infection were tested by MR-PRESSO for *p* = 0.004. The *p*-value for MR-PRESSO is 0.0273 after excluding the outlier SNP (rs16832436), suggesting no remaining outlier SNPs. At this point, MR Egger’s *P* (Q-statistic) = 0.0410, and IVW’s *P* (Q-statistic) = 0.0283. However, the MR Egger intercept test (*p* = 0.1229) showed no directional pleiotropy, indicating that heterogeneity is unlikely to affect the main estimates. The remaining MR analyses had *P* (Q-statistic) > 0.05, and the funnel plots of SNPs in IVW had a symmetrical distribution, indicating no significant heterogeneity (Supplementary Figure S2). The *p*-value of all MR Egger intercept tests was greater than 0.05, suggesting no directional pleiotropy of SNPs, indicating the high validity and robustness of the results of the MR analyses in this study. In addition, no significant individual SNPs were found to influence the association from leave-one-out analyses (Supplementary Figure S3). In conclusion, the forest plot shows that HSV, VZV, EBV, CMV, HHV-6, and HHV-7 were not causally associated with ALS (Figure 2).

**Table 1:** The causal effect of human herpes viruses (HHVs) and amyotrophic lateral sclerosis (ALS) by two-sample Mendelian Randomization (TSMR) and the sensitivity analysis results.

Forward MR								
Exposure	SNP	F	Mendelian randomization				Heterogeneity	
			Method	OR	95% confidence interval	*p*	Q	Q_P
HSV infections	69	>10	MR Egger	0.9761	0.9320–1.0221	0.3069	60.0225	0.7146
			Weighted median	0.9971	0.9620–1.0335	0.8739		
			IVW	0.9917	0.9685–1.0154	0.4886	60.6399	0.7250
			Simple mode	1.0402	0.9636–1.1228	0.3161		
			Weighted mode	0.9979	0.9401–1.0592	0.9443		
			MR-PRESSO			0.7357		
			Pleiotropy			0.4348		
HSV keratitis and keratoconjunctivitis	55	>10	MR Egger	0.9820	0.9539–1.0109	0.2248	61.9019	0.1882
			Weighted median	0.9847	0.9623–1.0077	0.1901		
			IVW	0.9897	0.9739–1.0059	0.2107	62.3818	0.2028
			Simple mode	1.0099	0.9578–1.0649	0.7167		
			Weighted mode	0.9836	0.9453–1.02340	0.4180		
			MR-PRESSO			0.2157		
			Pleiotropy			0.5243		
Anogenital HSV infection	56	>10	MR Egger	1.0419	0.9916–1.0947	0.1097	73.3438	0.0410
			Weighted median	1.0080	0.9767–1.0403	0.6206		
			IVW	1.0062	0.9826–1.0304	0.6081	76.6802	0.0283
			Simple mode	0.9611	0.8859–1.0427	0.3443		
			Weighted mode	0.9684	0.9071–1.0339	0.3402		
			MR-PRESSO			0.0273(Outlier: rs16832436)		
			Pleiotropy			0.1229		
VZV lgG	65	>10	MR Egger	1.0110	0.9809–1.0420	0.4820	71.9553	0.2056
			Weighted median	0.9994	0.9776–1.0217	0.9603		
			IVW	1.0003	0.9849–1.0160	0.9659	72.6867	0.2136
			Simple mode	0.9988	0.9475–1.0530	0.9657		
			Weighted mode	1.0024	0.9552–1.0519	0.9230		
			MR-PRESSO			0.2187		
			Pleiotropy			0.4266		
EBV	6	>10	MR Egger	0.8207	0.6170–1.0917	0.2463	3.0553	0.5486
			Weighted median	0.9753	0.8930–1.0653	0.5788		
			IVW	0.9509	0.8879–1.0183	0.1497	4.1400	0.5294
			Simple mode	0.8870	0.7663–1.0268	0.1692		
			Weighted mode	1.0097	0.8865–1.1499	0.8906		
			MR-PRESSO			0.5577		
			Pleiotropy			0.3565		
CMV lgG	7	>10	MR Egger	0.8688	0.7344–1.0279	0.1621	3.6783	0.5966
			Weighted median	0.9603	0.8553–1.0784	0.4938		
			IVW	0.9481	0.8680–1.0357	0.2374	5.1108	0.5297
			Simple mode	0.9595	0.8133–1.1321	0.6419		
			Weighted mode	0.9618	0.8194–1.1289	0.6505		
			MR-PRESSO			0.5770		
			Pleiotropy			0.2850		
HHV-6 lgG	5	>10	MR Egger	0.9718	0.8714–1.0838	0.6424	1.8228	0.6100
			Weighted median	0.9737	0.9262–1.0237	0.2976		
			IVW	0.9884	0.9486–1.0298	0.5765	1.9307	0.7485
			Simple mode	0.9688	0.9038–1.0385	0.4214		
			Weighted mode	0.9647	0.9003–1.0338	0.3663		
			MR-PRESSO			0.7317		
			Pleiotropy			0.7641		
HHV-7 lgG	6	>10	MR Egger	0.9906	0.8696–1.1284	0.8938	5.2634	0.2613
			Weighted median	1.0156	0.9777–1.0550	0.4242		
			IVW	0.9991	0.9693–1.0299	0.9557	5.2870	0.3819
			Simple mode	1.0232	0.9609–1.0897	0.5059		
			Weighted mode	1.0232	0.9644–1.0857	0.4810		
			MR-PRESSO			0.4127		
			Pleiotropy			0.8999		

### 3.3 Reverse MR

**Figure 3:**
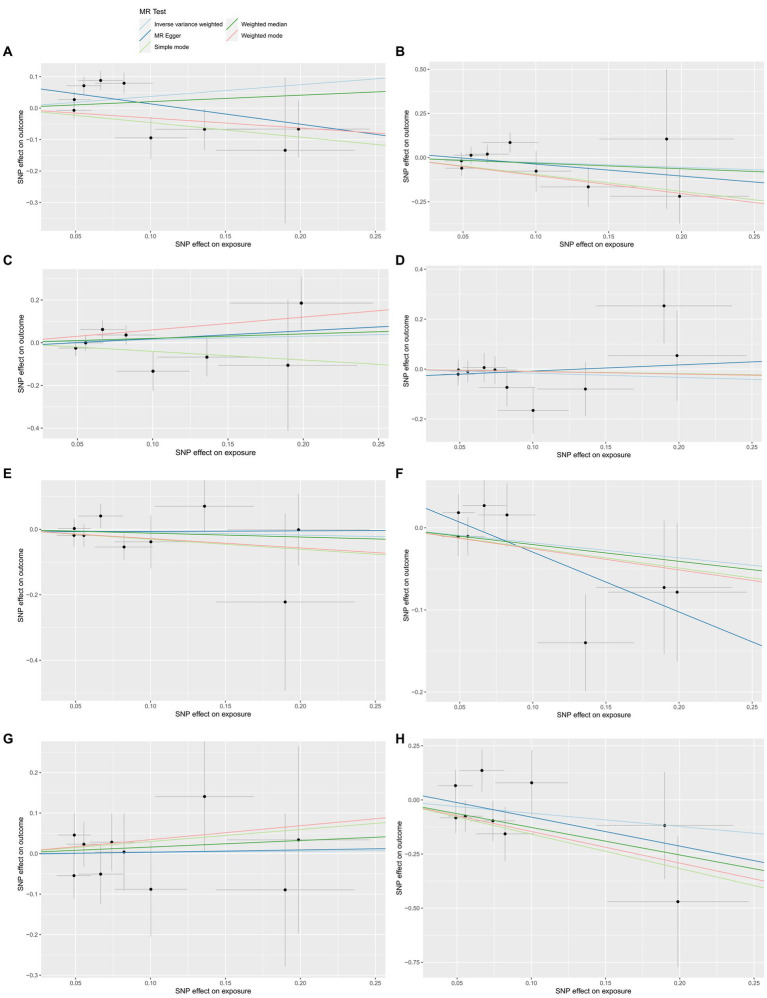
The reverse MR effect of ALS and HHVs. Scatter plots for highlighting the effect of ALS on HSV infections**(A)**, HSV keratitis and keratoconjunctivitis **(B)**, anogenital HSV infection **(C)**, VZV IgG **(D)**, EBV **(E)**, CMV IgG **(F)**, HHV-6 IgG **(G)**, HHV-7 IgG **(H)** using the MR-Egger, weighted median, IVW, simple mode and weighted mode methods.

**Figure 4:**
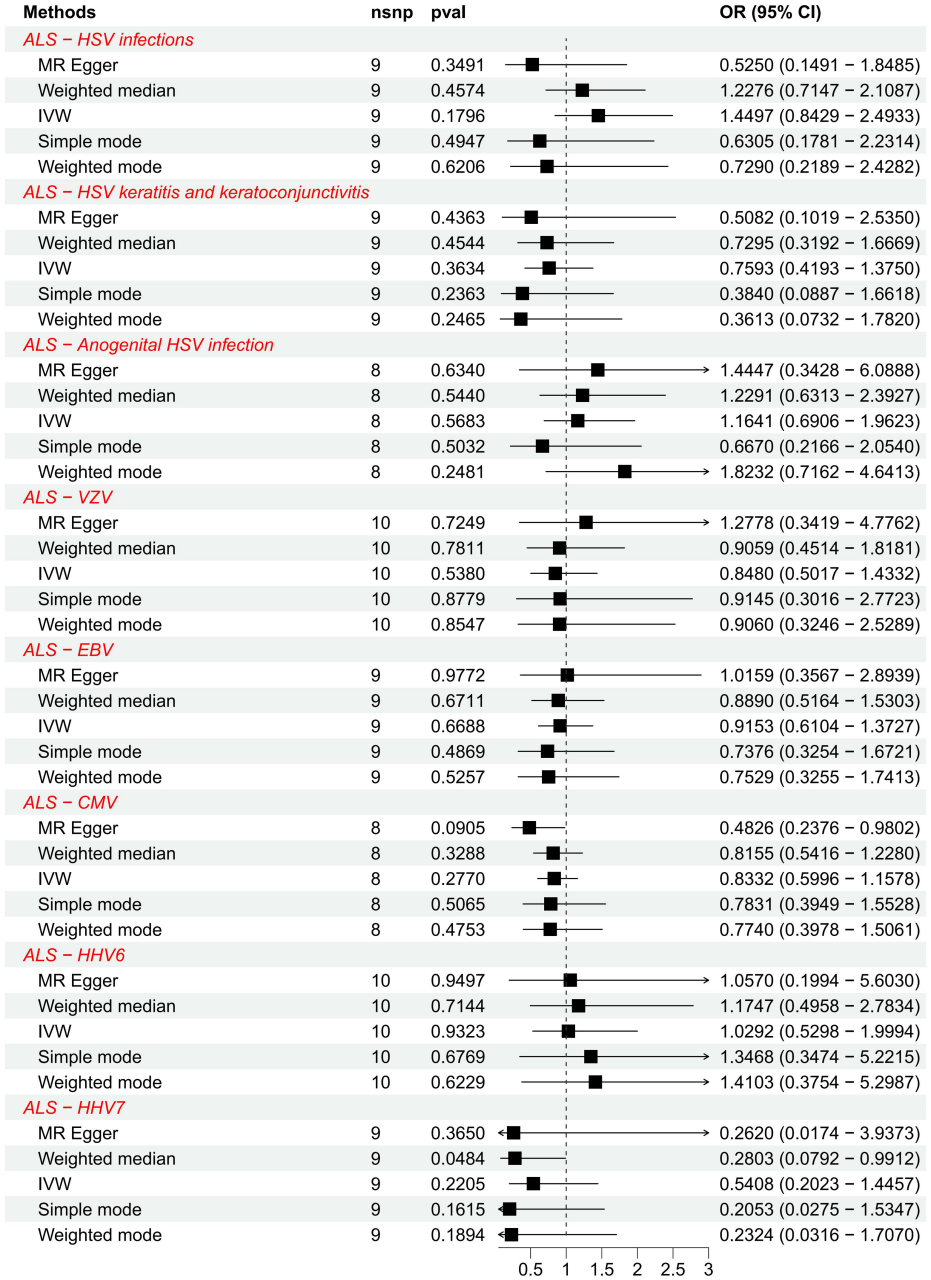
Forest plots of causal effect estimates in the reverse MR analysis. SNP, single-nucleotide polymorphism; ALS, amyotrophic lateral sclerosis; IVW, inverse variance weighted; OR, odds ratio; 95% CI, 95% confidence interval.

We further explored the causal association of ALS with HHVs and enumerated the results in Table 2. There was also no causal effect of ALS with HSV infections (OR = 1.4497; 95% CI: 0.8429–2.4933; *p* = 0.1796), HSV keratitis and keratoconjunctivitis (OR = 0.7593; 95% CI: 0.4193–1.3750; *p* = 0.3634), anogenital HSV infection (OR = 1.1641; 95% CI: 0.6906–1.9623; *p* = 0.5683), VZV IgG (OR = 0.8480; 95% CI: 0.5017–1.4332; *p* = 0.5380), EBV (OR = 0.9153; 95% CI: 0.6104–1.3727; *p* = 0.6688), CMV (OR = 0.8332; 95% CI: 0.5996–1.1578; *p* = 0.2770), HHV-6 IgG (OR = 1.0292; 95% CI: 0.5298–1.9994; *p* = 0.9323) and HHV-7 IgG (OR = 0.5408; 95% CI: 0.2023–1.4457; *p* = 0.2205) in the IVW analysis, which generally agreed with the results obtained from the remaining four MR analyses (Figure 3). Notably, the MR-PRESSO of the HSV infections dataset was tested for a *p*-value of 0.0130, but no outlier SNPs. At this point, MR Egger’s *P* (Q-statistic) = 0.0404, and IVW’s *P* (Q-statistic) = 0.0076. However, the MR Egger intercept test with a *p*-value of 0.1229 did not show directional pleiotropy, suggesting that heterogeneity is unlikely to affect the main estimates. MR-PRESSO test on the anogenital HSV infection dataset found a *p*-value of 0.0270, suggesting heterogeneity, and a *p*-value of 0.3623 for MR-PRESSO after excluding outlier SNPs (rs17524886), and did not identify other outlier SNPs. At this point, MR Egger’s *P* (Q-statistic) = 0.2497, and IVW’s *P* (Q-statistic) = 0.3346, indicating no heterogeneity. The MR-PRESSO test of the HHV-7 IgG dataset found a *p*-value of 0.0467, a *p*-value of 0.3500 after excluding outlier SNPs (rs4669231) and suggesting no other outlier SNPs, and a *P* (Q-statistic) = 0.2704 for MR Egger and *P* (Q-statistic) = 0.3289, indicating no heterogeneity. The *p*-value of the Q-statistic for the rest of the dataset was greater than 0.05, and the SNPs in the funnel plot of IVW were symmetrically distributed, indicating no significant heterogeneity (Supplementary Figure S4). All MR Egger intercept tests had *p*-values greater than 0.05, suggesting that the SNPs were free of directional pleiotropy, which suggests good validity and robustness of the MR analysis. In addition, no individual SNPs capable of driving causality between exposure and outcome were identified from leave-one-out analyses (Supplementary Figure S5). In conclusion, the forest plot shows that ALS was not causally associated with HSV, VZV, EBV, CMV, HHV-6, and HHV-7 (Figure 4).

**Table 2:** The causal effect of amyotrophic lateral sclerosis (ALS) and human herpes viruses (HHVs) by two-sample Mendelian Randomization (TSMR) and the sensitivity analysis results.

Reverse MR								
Outcome	SNP	F	Mendelian randomization				Heterogeneity	
			Method	OR	95% confidence interval	P	Q	Q_P
HSV infections	9	>10	MR Egger	0.5250	0.1491–1.8485	0.3491	14.6775	0.0404
			Weighted median	1.2276	0.7147–2.1087	0.4574		
			IVW	1.4497	0.8429–2.4933	0.1796	20.8434	0.0076
			Simple mode	0.6305	0.1781–2.2314	0.4947		
			Weighted mode	0.7290	0.2189–2.4282	0.6206		
			MR-PRESSO			0.0130 (No outlier)		
			pleiotropy			0.1301		
HSV keratitis and keratoconjunctivitis	9	>10	MR Egger	0.5082	0.1019–2.5350	0.4363	8.1388	0.3205
			Weighted median	0.7295	0.3192–1.6669	0.4544		
			IVW	0.7593	0.4193–1.3750	0.3634	8.4668	0.3893
			Simple mode	0.3840	0.0887–1.6618	0.2363		
			Weighted mode	0.3613	0.0732–1.7820	0.2465		
			MR-PRESSO			0.3510		
			pleiotropy			0.6118		
Anogenital HSV infection	8	>10	MR Egger	1.4447	0.3428–6.0888	0.6340	7.8441	0.2497
			Weighted median	1.2291	0.6313–2.3927	0.5440		
			IVW	1.1641	0.6906–1.9623	0.5683	7.9774	0.3346
			Simple mode	0.6670	0.2166–2.0540	0.5032		
			Weighted mode	1.8232	0.7162–4.6413	0.2481		
			MR-PRESSO			0.3623 (Outlier: rs17524886)		
			pleiotropy			0.7603		
VZV lgG	10	>10	MR Egger	1.2778	0.3419–4.7762	0.7249	7.0744	0.5286
			Weighted median	0.9059	0.4514–1.8181	0.7811		
			IVW	0.8480	0.5017–1.4332	0.5380	7.5159	0.5836
			Simple mode	0.9145	0.3016–2.7723	0.8779		
			Weighted mode	0.9060	0.3246–2.5289	0.8547		
			MR-PRESSO			0.5907		
			pleiotropy			0.5251		
EBV	9	>10	MR Egger	1.0159	0.3567–2.8939	0.9772	5.1470	0.6420
			Weighted median	0.8890	0.5164–1.5303	0.6711		
			IVW	0.9153	0.6104–1.3727	0.6688	5.1919	0.7369
			Simple mode	0.7376	0.3254–1.6721	0.4869		
			Weighted mode	0.7529	0.3255–1.7413	0.5257		
			MR-PRESSO			0.7260		
			pleiotropy			0.8383		
CMV lgG	8	>10	MR Egger	0.4826	0.2376–0.9802	0.0905	5.0916	0.5321
			Weighted median	0.8155	0.5416–1.2280	0.3288		
			IVW	0.8332	0.5996–1.1578	0.2770	7.9111	0.3405
			Simple mode	0.7831	0.3949–1.5528	0.5065		
			Weighted mode	0.7740	0.3978–1.5061	0.4753		
			MR-PRESSO			0.3553		
			pleiotropy			0.1441		
HHV-6 lgG	10	>10	MR Egger	1.0570	0.1994–5.6030	0.9497	4.3357	0.8256
			Weighted median	1.1747	0.4958–2.7834	0.7144		
			IVW	1.0292	0.5298–1.9994	0.9323	4.3369	0.8879
			Simple mode	1.3468	0.3474–5.2215	0.6769		
			Weighted mode	1.4103	0.3754–5.2987	0.6229		
			MR-PRESSO			0.8853		
			pleiotropy			0.9736		
HHV-7 lgG	9	>10	MR Egger	0.2620	0.0174–3.9373	0.3650	8.7596	0.2704
			Weighted median	0.2803	0.0792–0.9912	0.0484		
			IVW	0.5408	0.2023–1.4457	0.2205	9.1611	0.3289
			Simple mode	0.2053	0.0275–1.5347	0.1615		
			Weighted mode	0.2324	0.0316–1.7070	0.1894		
			MR-PRESSO			0.3500 (Outlier: rs4669231)		
			pleiotropy			0.5888		

## 4 Discussion

Neurodegenerative diseases can damage and degenerate neurons in the CNS and peripheral nervous system, resulting in severe loss of memory, behavior, and sensory and motor functions due to the inability of the neurons to renew and regenerate. Classic NDDs include Parkinson’s disease, Alzheimer’s disease, and ALS, which impose an enormous global burden (Wilson et al., 2023). Although the pathogenic mechanism of ALS is unclear, it is believed that it may be due to oxidative stress, apoptosis, mitochondrial dysfunction, axonal degeneration, neuroinflammation, and viruses (Sever et al., 2022).

Among HHVs, the most well-known HSV can infect neurons and reach the CNS through retrograde axonal transport, ultimately leading to diseases such as encephalomyelitis, and several studies have shown that HSV is closely related to NDDs (Carneiro et al., 2022). Primary infection with VZV causes varicella and can latently infect neurons. When the body is immunocompromised or aged, VZV is reactivated and causes a zoster. In addition, VZV can cause neurological syndromes such as myelitis and segmental motor paralysis (Gershon et al., 2015). EBV is most commonly associated with infectious mononucleosis, while primary or latent infections are mostly associated with neurological disorders, such as encephalomyelitis (Andersen et al., 2023). CMV can exhibit tropism for neural stem cells and cause multiple spinal cord radiculitis (Kleinschmidt-DeMasters and Gilden, 2001; Kamte et al., 2021). The viral load of HHV-6 is associated with increased central nervous system demyelination (Lucas et al., 2023). The damage to the CNS system by HHV-7 has also been reported (Li et al., 2022).

However, do these HHVs increase the risk of ALS? Earlier studies reported that chronic viral infections play an important role in the pathogenesis of ALS, and antibody titers to HSV-1 were significantly increased in the sera of ALS patients (Irkeç et al., 1989). In contrast, studies have reported that significant elevations of HSV-1, HSV-2, and VZV antibodies were not detected in the sera of ALS patients (Catalano, 1972). In a mouse model of latent HSV-2 infection, it was found that HSV-2-induced spinal cord inflammation, although similar to that of ALS patients, was insufficient to induce the characteristic changes in the pathology of ALS (Cabrera et al., 2020). In addition, it has been reported that HSV-1, EBV, CMV, and HHV-6 can cause motor neuron degeneration by activating endogenous retroviruses, thereby inducing the expression of the envelope glycoprotein HERV-K in ALS (Medina et al., 2017; Mayer et al., 2018). However, a subsequent study questioned this view due to their failure to detect highly expressed HERV-K RNA in ALS (Garson et al., 2019). Therefore, a causal association between HHVs and ALS cannot be stated based on the available evidence, and our systematic MR analyses can contribute to related studies.

Thus, studies on the relationship between human herpesviruses and ALS are conflicting, and there are few reports of a causal association between them. We concluded that there is no evidence to support a causal association between HHVs (HSV, VZV, EBV, CMV, HHV-6, and HHV-7) and ALS using bidirectional TSMR based on a sizable sample of GWAS data, implying that observational studies in which HHVs and ALS are associated may be due to confounding factors such as environment or shared genetic structure.

Our bidirectional TSMR study focused on the causal relationship between HHVs and ALS for the first time. This study has several strengths. Firstly, we are not limited to studying HSV and ALS but have expanded to study the causal relationship between multiple HHVs and ALS. Secondly, a bidirectional TSMR analysis reduces bias from confounding factors and excludes the effects caused by reverse causality. Of course, this study has some limitations. First, the significance threshold was relaxed from 5 × 10 ^−8^ to 5 × 10 ^−5^ because the number of IVs was so small. Distortion caused by weak instruments is possible, even though the resulting IVs were all defined as strong instrumental variables after calculating the F statistic (*F* > 10). Second, although sensitivity analyses of MR indicate robustness among SNPs, there is still the possibility of residual heterogeneity. Finally, we only analyzed GWAS data using European ancestry, and results should be interpreted with caution when applied to other populations.

## 5 Conclusion

We have shown no causal association between genetically predicted human herpes viruses (HSV, VZV, EBV, CMV, HHV-6, and HHV-7) and ALS based on Mendelian randomization analysis of currently relevant GWAS data. The associations observed in epidemiological studies may be partly attributable to shared genetic structure or environmental confounders, and we could devote more time and money to studies of other environmental factors associated with ALS and genetic structure.

## Data availability statement

The original contributions presented in the study are included in the article/Supplementary material, further inquiries can be directed to the corresponding authors.

## Author contributions

QZ: Writing – original draft. DW: Writing – original draft. RL: Writing – original draft. YC: Writing – original draft. HH: Writing – original draft. ZX: Writing – review & editing. CZ: Writing – review & editing. WX: Writing – review & editing.

## Footnotes

^1^ https://r9.finngen.fi/

^2^ https://www.ebi.ac.uk/gwas/home
