# Supplementary material for: Mendelian randomization analysis suggests no associations of human herpes viruses with amyotrophic lateral sclerosis
Source: Front Neurosci. 2023 Dec 12;17:1299122. doi: 10.3389/fnins.2023.1299122 (PMC10754516; doi:10.3389/fnins.2023.1299122)
Supplement: Supplementary file 1 [file Data_Sheet_1.doc]

Supplementary Material

**Figure S1.** Assumptions in MR analysis.

**Figure S2.** Funnel plot of the forward MR analysis of HSV infections (A), HSV keratitis and keratoconjunctivitis (B), anogenital HSV infection (C), VZV IgG (D), EBV (E), CMV IgG (F), HHV-6 IgG (G), HHV-7 IgG (H) causal association on ALS.

**Figure S3.** Leave-on-out analysis to evaluate whether every single SNP was driving the causal association of HSV infections (A), HSV keratitis and keratoconjunctivitis (B), anogenital HSV infection (C), VZV IgG (D), EBV(E), CMV IgG(F), HHV-6 IgG (G), HHV-7 IgG (H) on ALS in the forward MR analysis.

**Figure S4.** Funnel plot of the reverse MR analysis of ALS on HSV infections (A), HSV keratitis and keratoconjunctivitis (B), anogenital HSV infection (C), VZV IgG (D), EBV (E), CMV IgG (F), HHV-6 IgG (G), and HHV-7 IgG (H).

**Figure S5.** Leave-on-out analysis to evaluate whether every single SNP was driving the causal association of ALS on HSV infections (A), HSV keratitis and keratoconjunctivitis (B), anogenital HSV infection(C), VZV IgG (D), EBV (E), CMV IgG (F), HHV-6 IgG (G), and HHV-7 IgG (H) in the reverse MR analysis.

**Table S1.** Details of the genome-wide association studies and datasets used in this study.

**Table S2.** Detailed information on the instrumental variables (IVs) for HSV infections and ALS in the forward MR analysis.

**Table S3.** Detailed information on the instrumental variables (IVs) for HSV keratitis, keratoconjunctivitis, and ALS in the forward MR analysis.

**Table S4.** Detailed information on the instrumental variables (IVs) for Anogenital HSV infection and ALS in the forward MR analysis.

**Table S5.** Detailed information on the instrumental variables (IVs) for VZV IgG and ALS in the forward MR analysis.

**Table S6.** Detailed information on the EBV and ALS instrumental variables (IVs) in the forward MR analysis.

**Table S7.** Detailed information on the CMV IgG and ALS instrumental variables (IVs) in the forward MR analysis.

**Table S8.** Detailed information on the instrumental variables (IVs) for HHV-6 IgG and ALS in the forward MR analysis.

**Table S9.** Detailed information on the instrumental variables (IVs) for HHV-7 IgG and ALS in the forward MR analysis.

**Table S10.** Detailed information on the instrumental variables (IVs) for ALS and HSV infections in the reverse MR analysis.

**Table S11.** Detailed information on the instrumental variables (IVs) for ALS and HSV keratitis and keratoconjunctivitis in the reverse MR analysis.

**Table S12.** Detailed information on the instrumental variables (IVs) for ALS and anogenital HSV infection in the reverse MR analysis.

**Table S13.** Detailed information on the ALS and VZV IgG instrumental variables (IVs) in the reverse MR analysis.

**Table S14.** Detailed information on ALS and EBV instrumental variables (IVs) in the reverse MR analysis.

**Table S15.** Detailed information on the ALS and CMV IgG instrumental variables (IVs) in the reverse MR analysis.

**Table S16.** Detailed information on the instrumental variables (IVs) for ALS and HHV-6 IgG in the reverse MR analysis.

**Table S17.** Detailed information on the instrumental variables (IVs) for ALS and HHV-7 IgG in the reverse MR analysis.

**
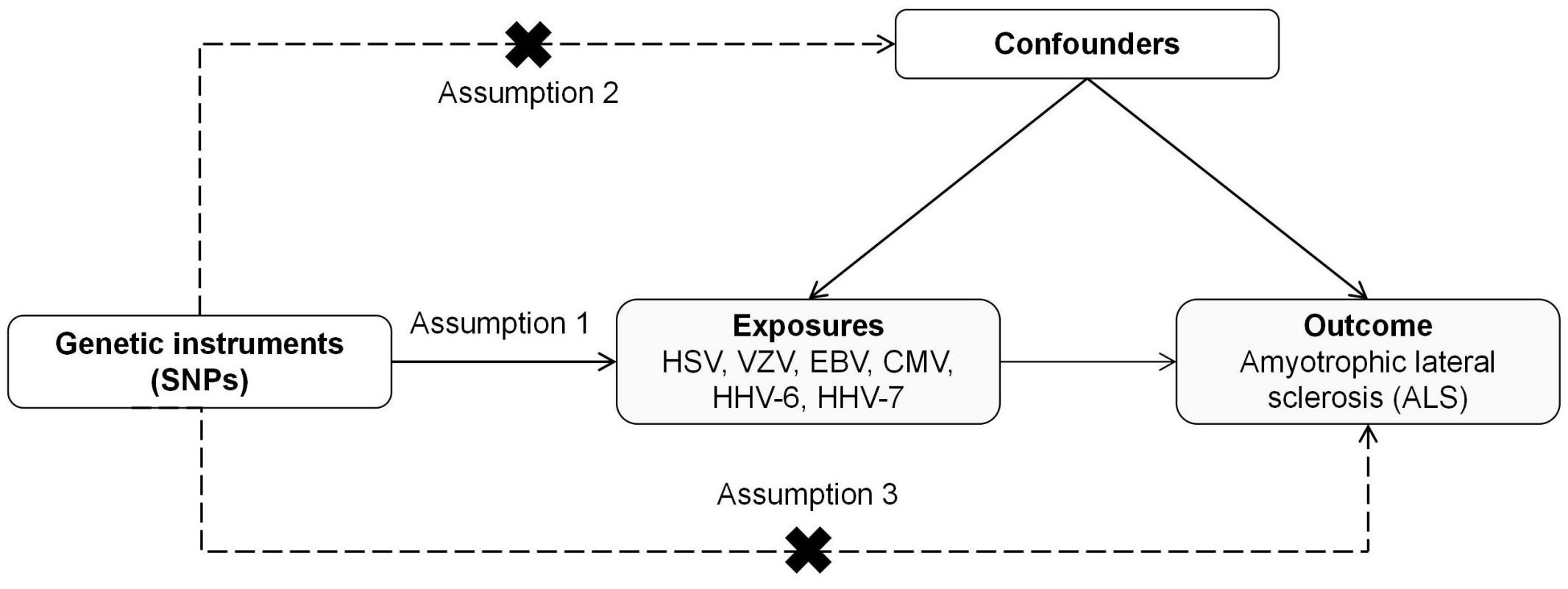
**

**Figure S1. Assumptions in MR analysis.** Three assumptions of MR are as follows: (1) Assumption 1: Genetic instruments must be significantly associated with the exposure; (2) Assumption 2: Genetic instruments are independent of confounders; (3) Assumption 3: Genetic instruments affect the outcome only via exposure.


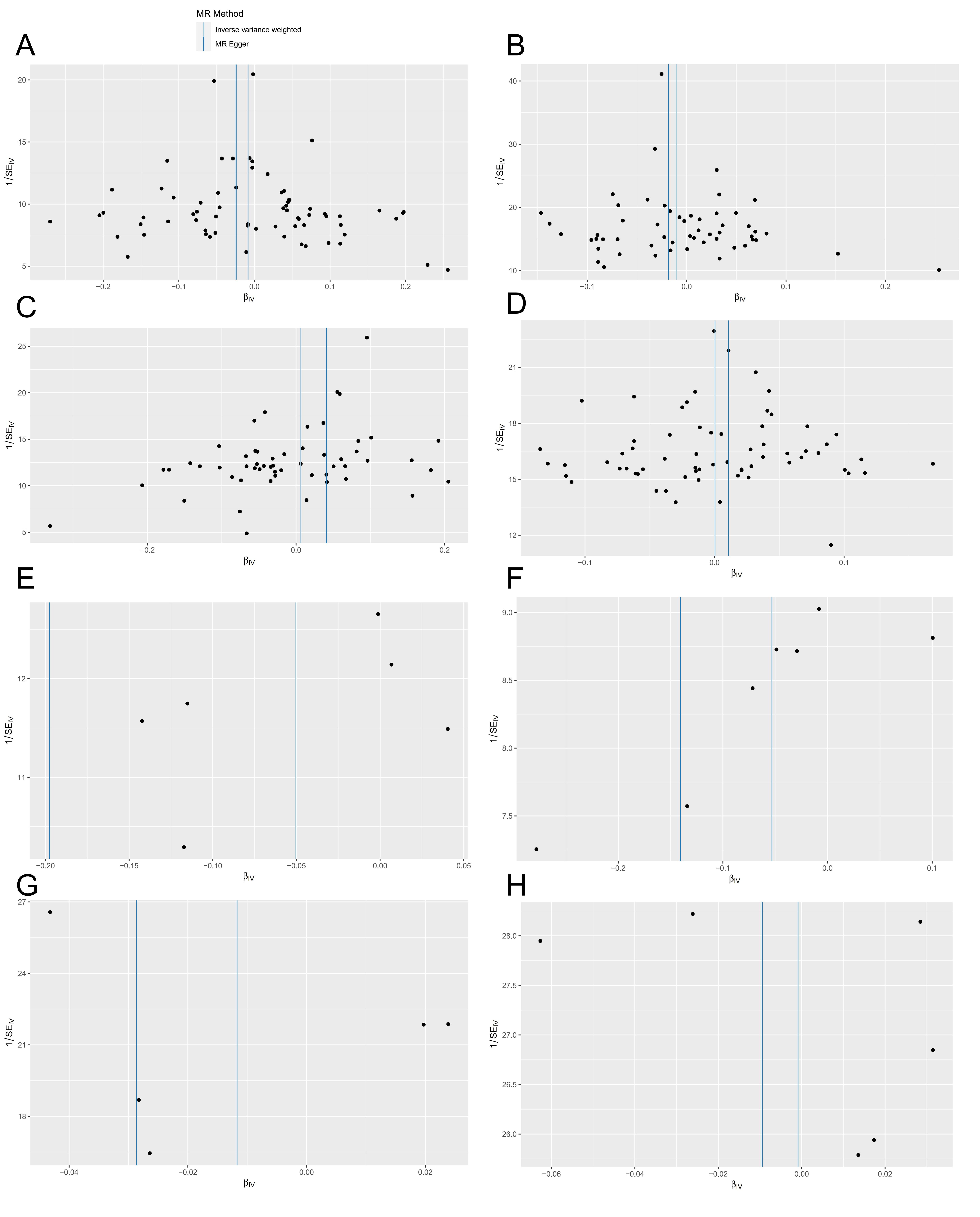


**Figure S2.** Funnel plot of the forward MR analysis of HSV infections (A), HSV keratitis and keratoconjunctivitis (B), anogenital HSV infection (C), VZV IgG (D), EBV (E), CMV IgG (F), HHV-6 IgG (G), HHV-7 IgG (H) causal association on ALS.


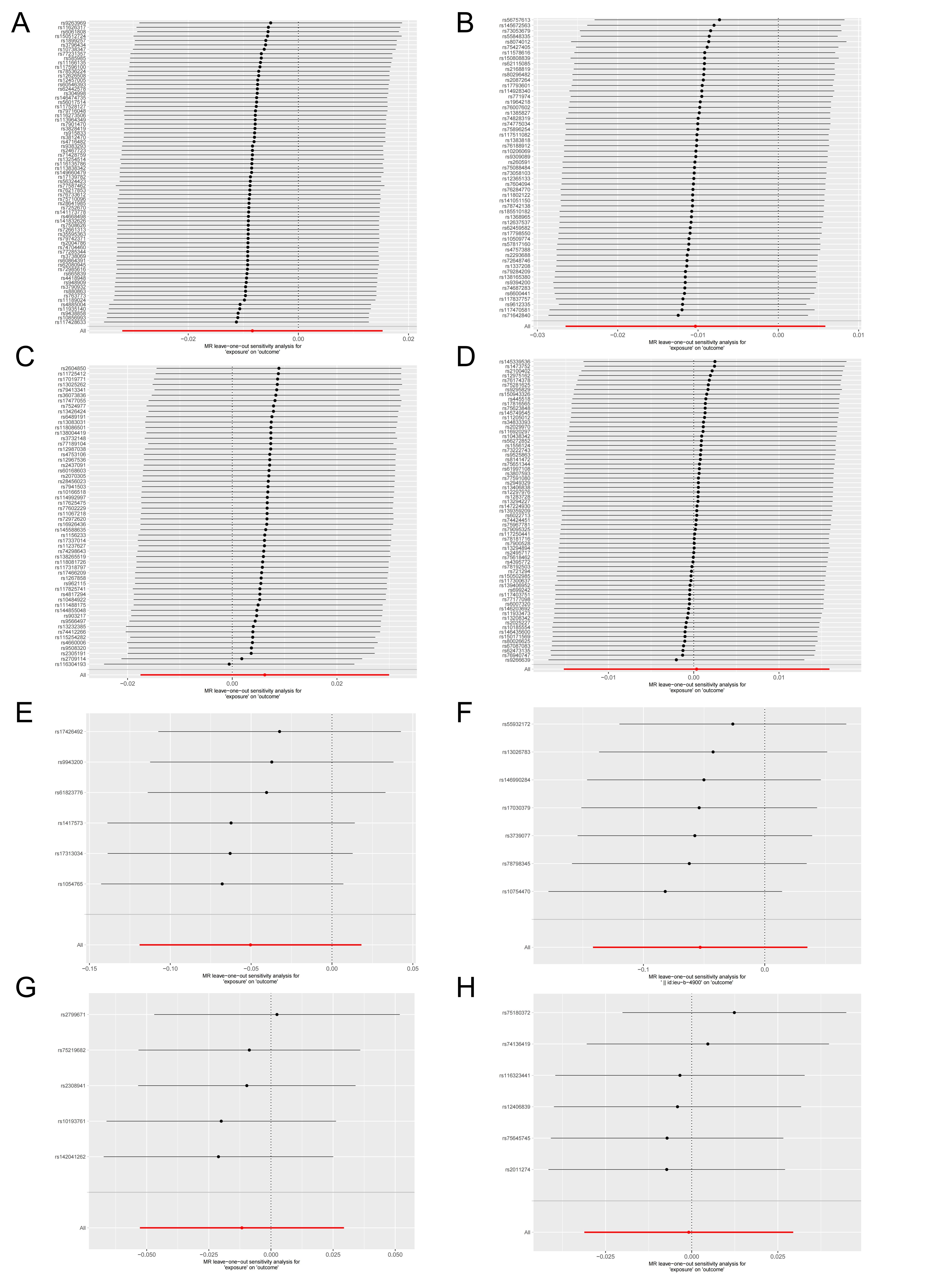


**Figure S3.** Leave-on-out analysis to evaluate whether every single SNP was driving the causal association of HSV infections (A), HSV keratitis and keratoconjunctivitis (B), anogenital HSV infection (C), VZV IgG (D), EBV(E), CMV IgG(F), HHV-6 IgG (G), HHV-7 IgG (H) on ALS in the forward MR analysis.


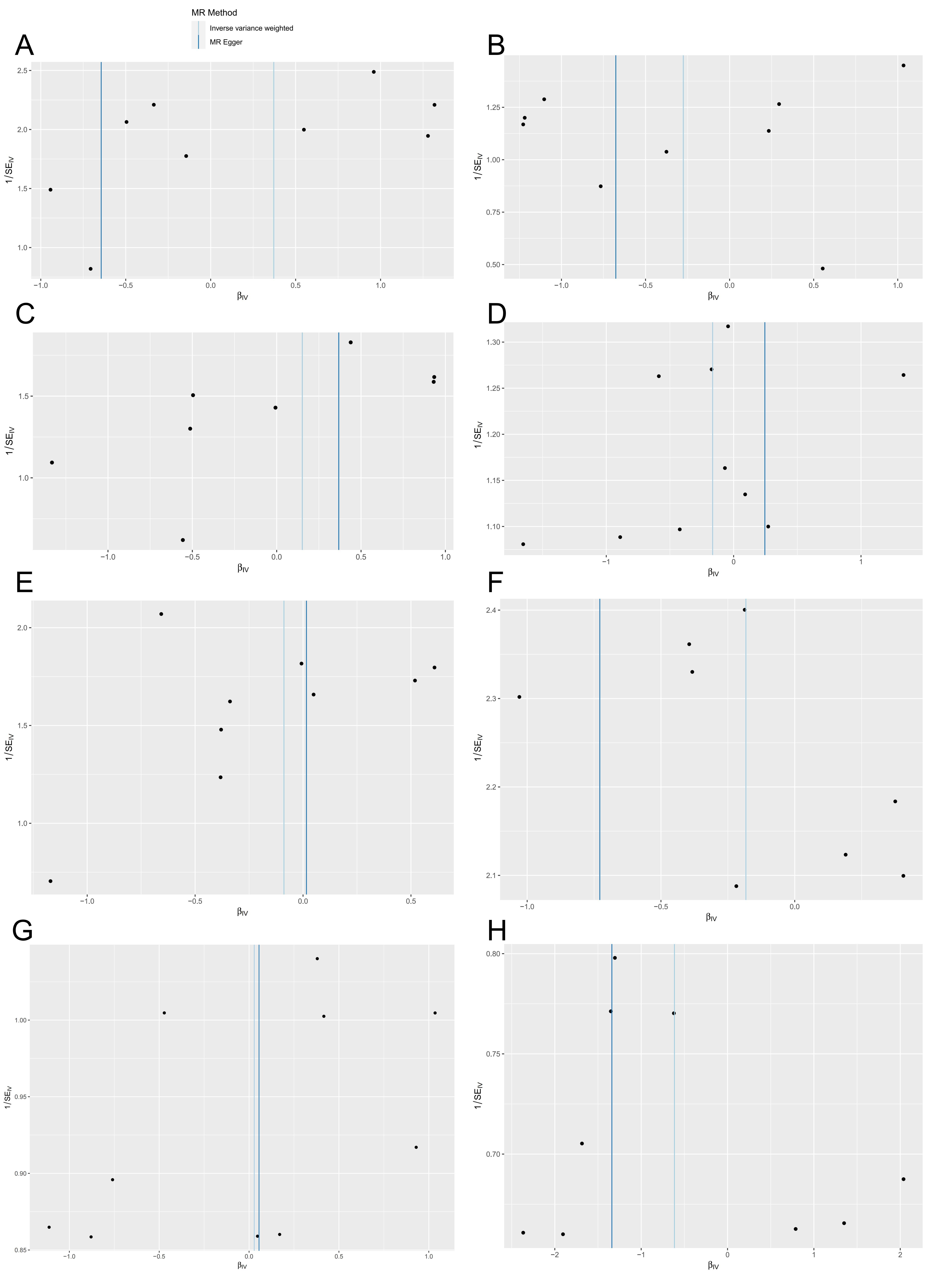


**Figure S4.** Funnel plot of the reverse MR analysis of ALS on HSV infections (A), HSV keratitis and keratoconjunctivitis (B), anogenital HSV infection (C), VZV IgG (D), EBV (E), CMV IgG (F), HHV-6 IgG (G), and HHV-7 IgG (H).


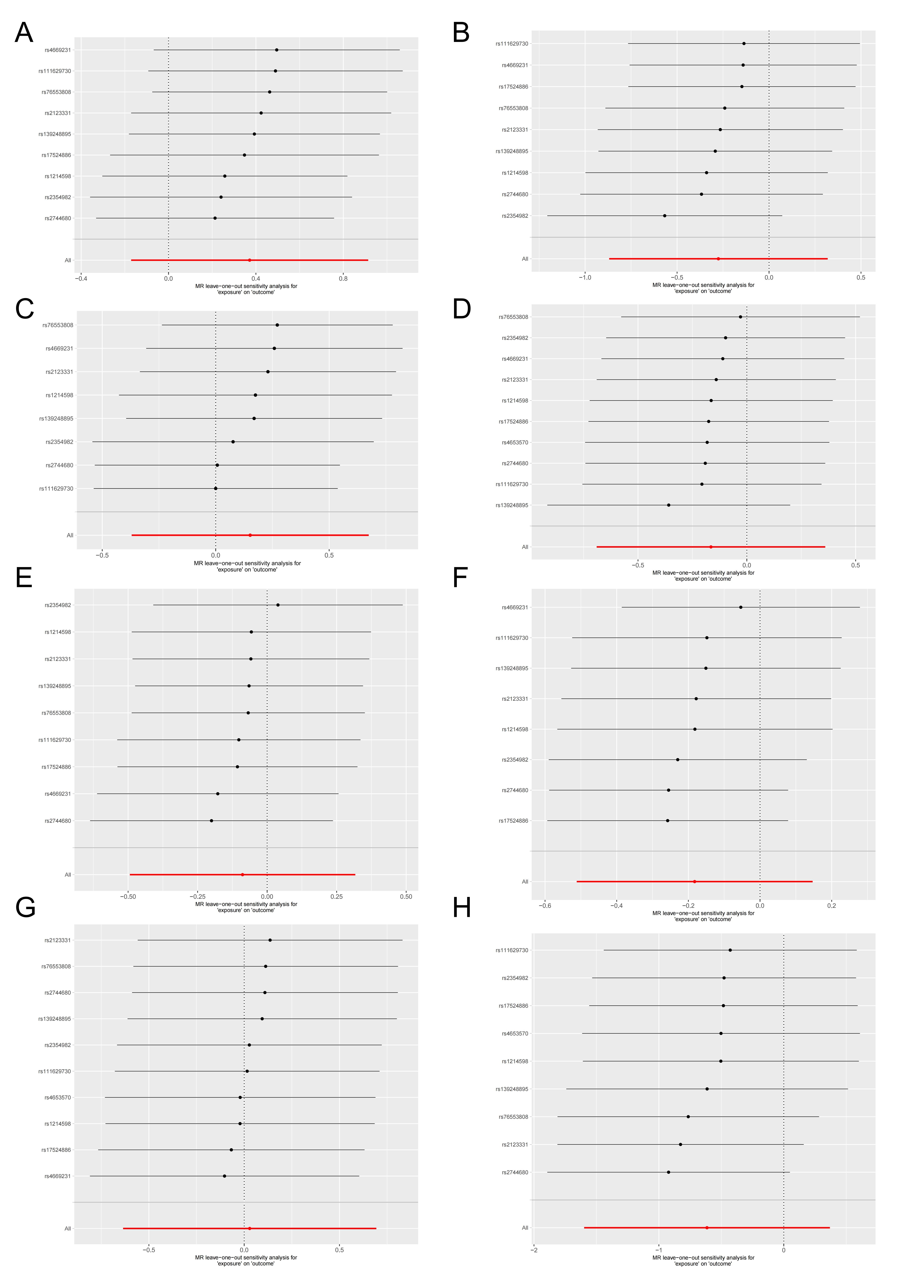


**Figure S5.** Leave-on-out analysis to evaluate whether every single SNP was driving the causal association of ALS on HSV infections (A), HSV keratitis and keratoconjunctivitis (B), anogenital HSV infection(C), VZV IgG (D), EBV (E), CMV IgG (F), HHV-6 IgG (G), and HHV-7 IgG (H) in the reverse MR analysis.

**Table S1. Details of the genome-wide association studies and datasets used in this study.**

| **Exposure and outcome** | **Source** | **GWAS ID** | **Sample size** | **Cases** | **Controls** | **SNP** | **Ancestry** | **Access Link** |
| --- | --- | --- | --- | --- | --- | --- | --- | --- |
| HSV infections | FinnGen (r9) | finn-b-AB1_HERPES_SIMPLEX | 213,451 | 1,595 | 211,856 | 16,380,457 | European | https://r9.finngen.fi/ |
| HSV keratitis and keratoconjunctivitis | FinnGen (r9) | finn-b-H7_HERPESKERATITIS | 209,860 | 573 | 209,287 | 16,380,429 | European | https://r9.finngen.fi/ |
| Anogenital HSV infection | FinnGen (r9) | finn-b-AB1_ANOGENITAL_HERPES_SIMPLEX | 213,843 | 891 | 212,952 | 16,380,455 | European | https://r9.finngen.fi/ |
| VZV IgG | Butler-Laporte G | GCST90006928 | 8,735 | - | - | 25,472,218 | European | https://www.ebi.ac.uk/gwas/home |
| EBV | FinnGen (r9) | finn-b-AB1_EBV | 214,904 | 1,238 | 213,666 | 16,380,461 | European | https://r9.finngen.fi/ |
| CMV IgG | Chong A | ieu-b-4900 | 5,010 | - | - | 7,002,835 | European | https://gwas.mrcieu.ac.uk/ |
| HHV-6 IgG | Butler-Laporte G | GCST90006902 | 8,735 | - | - | 25,472,218 | European | https://www.ebi.ac.uk/gwas/home |
| HHV-7 IgG | Butler-Laporte G | GCST90006908 | 8,735 | - | - | 25,472,218 | European | https://www.ebi.ac.uk/gwas/home |
| ALS | van Rheenen W | GCST90027164 | 138,086 | 27,205 | 110,881 | 10,461,755 | European | https://www.ebi.ac.uk/gwas/ |

**Table S2. Detailed information on the instrumental variables (IVs) for HSV infections and ALS in the forward MR analysis.**

| **SNP** | **EA** | **OA** | **Exposure (HSV infections)** | | | | **Outcome (ALS)** | | | |
| --- | --- | --- | --- | --- | --- | --- | --- | --- | --- | --- |
|  |  |  | **beta** | **se** | **P** | **eaf** | **beta** | **se** | **P** | **eaf** |
| rs10738347 | C | G | 0.13273 | 0.0275784 | 1.49E-06 | 0.249836 | -0.0163 | 0.0118 | 0.1694 | 0.3118 |
| rs10856993 | C | T | 0.103908 | 0.0244863 | 2.20E-05 | 0.518708 | 0.0205 | 0.0111 | 0.06353 | 0.5037 |
| rs11166135 | G | A | 0.153443 | 0.0373855 | 4.05E-05 | 0.111124 | -0.0231 | 0.0183 | 0.207 | 0.1049 |
| rs11189024 | T | C | -0.144228 | 0.0349002 | 3.59E-05 | 0.158067 | -0.0163 | 0.016 | 0.3069 | 0.1572 |
| rs113838342 | T | C | 0.788806 | 0.191132 | 3.67E-05 | 0.00261531 | -0.0023 | 0.061 | 0.9697 | 0.0114 |
| rs113964349 | A | G | 0.178847 | 0.0403915 | 9.52E-06 | 0.0914463 | -0.0116 | 0.0227 | 0.6095 | 0.0625 |
| rs116135786 | A | G | 0.280463 | 0.061619 | 5.32E-06 | 0.035233 | 6.00E-04 | 0.035 | 0.9854 | 0.0309 |
| rs11626317 | A | G | -0.405951 | 0.085183 | 1.88E-06 | 0.0274886 | 0.0468 | 0.0301 | 0.1195 | 0.0395 |
| rs116273506 | A | G | 0.271639 | 0.0655184 | 3.38E-05 | 0.0315204 | -0.0125 | 0.0279 | 0.6552 | 0.0466 |
| rs117428633 | G | A | 1.0208 | 0.250707 | 4.67E-05 | 0.00134854 | 0.0777 | 0.0675 | 0.2499 | 0.008 |
| rs117528127 | C | T | -0.524611 | 0.110174 | 1.92E-06 | 0.0174775 | 0.0252 | 0.0481 | 0.6002 | 0.0146 |
| rs117596100 | T | C | 0.170078 | 0.0416302 | 4.40E-05 | 0.0859492 | -0.0308 | 0.0231 | 0.1818 | 0.0609 |
| rs11935140 | C | T | -0.122237 | 0.0286268 | 1.95E-05 | 0.253358 | -0.0202 | 0.0129 | 0.1176 | 0.2463 |
| rs12457005 | C | T | 0.29529 | 0.0587016 | 4.90E-07 | 0.0383833 | -0.0127 | 0.0216 | 0.5549 | 0.0778 |
| rs12626508 | C | G | -0.448129 | 0.104652 | 1.85E-05 | 0.0185145 | 0.0654 | 0.0595 | 0.272 | 0.0102 |
| rs13254514 | G | A | -0.202815 | 0.0459026 | 9.94E-06 | 0.0866272 | 0.0013 | 0.0148 | 0.9326 | 0.163 |
| rs141173778 | T | C | -0.160415 | 0.0388778 | 3.69E-05 | 0.122811 | -0.0069 | 0.0169 | 0.6843 | 0.1342 |
| rs141832626 | A | C | -0.392021 | 0.0966514 | 4.99E-05 | 0.0211867 | -0.0384 | 0.0571 | 0.5014 | 0.0133 |
| rs146474735 | G | A | 0.332848 | 0.0819143 | 4.84E-05 | 0.0186898 | -0.0558 | 0.0579 | 0.335 | 0.0118 |
| rs149660479 | G | T | -0.690904 | 0.162543 | 2.13E-05 | 0.00899136 | 0.002 | 0.0514 | 0.9686 | 0.0131 |
| rs150512724 | A | G | -0.987704 | 0.222265 | 8.84E-06 | 0.00567538 | 0.0527 | 0.0496 | 0.2878 | 0.0143 |
| rs17139782 | G | T | -0.100722 | 0.0247308 | 4.65E-05 | 0.587841 | -0.0028 | 0.0123 | 0.8212 | 0.6489 |
| rs1899257 | G | A | 0.104122 | 0.0247344 | 2.56E-05 | 0.530368 | -0.0208 | 0.0112 | 0.06461 | 0.5306 |
| rs2004786 | T | G | -0.118916 | 0.0262753 | 6.02E-06 | 0.33131 | -0.0053 | 0.0117 | 0.6521 | 0.3405 |
| rs2467723 | A | G | 0.112386 | 0.0273601 | 4.00E-05 | 0.709145 | -0.001 | 0.0136 | 0.9403 | 0.7705 |
| rs28641985 | A | G | 0.116726 | 0.0268756 | 1.40E-05 | 0.271678 | 0.0063 | 0.0142 | 0.6601 | 0.1844 |
| rs304998 | A | G | -0.111774 | 0.0269881 | 3.45E-05 | 0.726971 | 0.0085 | 0.0119 | 0.477 | 0.691 |
| rs35595363 | C | G | -0.341274 | 0.0794812 | 1.76E-05 | 0.0302792 | -0.0197 | 0.0385 | 0.6091 | 0.0295 |
| rs3738069 | T | A | 0.142717 | 0.0318542 | 7.45E-06 | 0.166475 | 0.0065 | 0.0138 | 0.6378 | 0.1955 |
| rs3790932 | T | C | -0.102034 | 0.0246232 | 3.42E-05 | 0.457942 | -0.0097 | 0.0113 | 0.3901 | 0.422 |
| rs3796434 | A | G | -0.100998 | 0.0245053 | 3.76E-05 | 0.475976 | 0.0207 | 0.0111 | 0.06167 | 0.4792 |
| rs3812470 | G | A | -0.158869 | 0.0381849 | 3.18E-05 | 0.126713 | 0.0082 | 0.0207 | 0.692 | 0.0762 |
| rs3828419 | C | T | 0.426938 | 0.092224 | 3.67E-06 | 0.0136501 | -0.0274 | 0.0564 | 0.6263 | 0.0183 |
| rs4418948 | A | T | -0.159164 | 0.0390878 | 4.66E-05 | 0.120309 | -0.019 | 0.0211 | 0.3686 | 0.0811 |
| rs4668498 | G | T | 0.109559 | 0.0259969 | 2.51E-05 | 0.650468 | 0.0046 | 0.0111 | 0.6791 | 0.472 |
| rs4716482 | C | A | -0.128065 | 0.0244759 | 1.67E-07 | 0.518843 | 0.0031 | 0.0113 | 0.7859 | 0.5312 |
| rs4885004 | G | A | 0.117364 | 0.0289187 | 4.94E-05 | 0.222863 | 0.022 | 0.0133 | 0.09714 | 0.2597 |
| rs56017514 | G | A | -0.148077 | 0.0361177 | 4.13E-05 | 0.143927 | 0.0114 | 0.017 | 0.5008 | 0.1348 |
| rs56324423 | A | G | 0.190406 | 0.0467297 | 4.61E-05 | 0.0655603 | 0.0075 | 0.0258 | 0.7723 | 0.0511 |
| rs585985 | G | T | 0.186244 | 0.0454904 | 4.24E-05 | 0.912655 | -0.0199 | 0.0177 | 0.2611 | 0.8906 |
| rs60546393 | T | C | 0.327496 | 0.0806497 | 4.89E-05 | 0.0189379 | -0.0233 | 0.0324 | 0.4718 | 0.031 |
| rs6061808 | G | C | 0.130632 | 0.0280365 | 3.17E-06 | 0.240092 | -0.0353 | 0.0152 | 0.0199 | 0.2698 |
| rs60864391 | T | C | -0.263423 | 0.0648717 | 4.89E-05 | 0.0429953 | -0.0122 | 0.0255 | 0.6307 | 0.0478 |
| rs62080945 | T | G | 0.21345 | 0.0525964 | 4.94E-05 | 0.0507471 | 0.0084 | 0.0193 | 0.6648 | 0.0871 |
| rs62442578 | T | C | -0.200265 | 0.0441421 | 5.71E-06 | 0.0935134 | 0.0162 | 0.0218 | 0.4567 | 0.0732 |
| rs665839 | C | T | 0.113099 | 0.0270076 | 2.82E-05 | 0.27074 | 0.0082 | 0.0124 | 0.5076 | 0.2947 |
| rs71428759 | G | T | 0.322959 | 0.0720334 | 7.34E-06 | 0.0242308 | -0.0027 | 0.0385 | 0.9433 | 0.0256 |
| rs7252670 | G | T | 0.110138 | 0.0256581 | 1.77E-05 | 0.330415 | 0.0042 | 0.0114 | 0.7113 | 0.3982 |
| rs72661313 | T | C | 0.196228 | 0.0468151 | 2.77E-05 | 0.0655024 | 0.0115 | 0.0223 | 0.6066 | 0.072 |
| rs72985616 | C | A | -0.286598 | 0.0684868 | 2.86E-05 | 0.0399417 | -0.0656 | 0.0562 | 0.2432 | 0.0133 |
| rs74704460 | G | A | 0.362378 | 0.0762866 | 2.03E-06 | 0.0212182 | 0.0411 | 0.0532 | 0.4397 | 0.0158 |
| rs7508626 | A | G | 0.126206 | 0.0305408 | 3.59E-05 | 0.187319 | 0.0083 | 0.0152 | 0.5821 | 0.1746 |
| rs75710096 | G | A | 0.253334 | 0.052802 | 1.60E-06 | 0.0485965 | 0.0044 | 0.0204 | 0.8289 | 0.0817 |
| rs76217853 | G | A | 0.415299 | 0.0967413 | 1.76E-05 | 0.0124937 | 0.0259 | 0.0615 | 0.673 | 0.0095 |
| rs763773 | T | G | -0.109614 | 0.0267193 | 4.09E-05 | 0.313483 | -0.0102 | 0.0119 | 0.3921 | 0.3067 |
| rs76733612 | T | C | 0.37627 | 0.086065 | 1.23E-05 | 0.0159907 | 0.0255 | 0.0569 | 0.6541 | 0.0099 |
| rs77231357 | G | A | 0.222173 | 0.0493916 | 6.85E-06 | 0.0590823 | -0.0326 | 0.0249 | 0.1908 | 0.0625 |
| rs77285344 | T | C | -0.34393 | 0.0823493 | 2.96E-05 | 0.0276264 | -0.0879 | 0.0732 | 0.2301 | 0.0071 |
| rs77587462 | G | C | -0.664702 | 0.154277 | 1.64E-05 | 0.009661 | 0.0012 | 0.0325 | 0.97 | 0.0293 |
| rs78536224 | G | C | 0.142761 | 0.0317324 | 6.83E-06 | 0.16735 | -0.0163 | 0.0166 | 0.3248 | 0.1332 |
| rs7901470 | G | A | 0.137694 | 0.0337131 | 4.42E-05 | 0.144638 | -0.007 | 0.0153 | 0.6484 | 0.1734 |
| rs79716048 | G | C | 0.541379 | 0.13168 | 3.93E-05 | 0.00652187 | -0.0154 | 0.0396 | 0.6973 | 0.0295 |
| rs79742371 | A | G | 0.432985 | 0.104187 | 3.24E-05 | 0.0109877 | 0.0156 | 0.0396 | 0.6944 | 0.0227 |
| rs880863 | A | G | 0.130624 | 0.030445 | 1.78E-05 | 0.786629 | 0.0149 | 0.0157 | 0.3425 | 0.8436 |
| rs915833 | C | T | -0.209162 | 0.0500862 | 2.97E-05 | 0.944287 | 0.0123 | 0.0284 | 0.6645 | 0.9516 |
| rs9263969 | T | C | -0.141794 | 0.0288529 | 8.91E-07 | 0.248506 | 0.0267 | 0.0127 | 0.03518 | 0.2454 |
| rs9383293 | G | A | 0.13008 | 0.0310854 | 2.86E-05 | 0.186526 | -0.0014 | 0.0212 | 0.9461 | 0.105 |
| rs9438858 | A | C | -0.104926 | 0.0247741 | 2.28E-05 | 0.444311 | -0.0206 | 0.0113 | 0.06913 | 0.4268 |
| rs948909 | A | G | 0.145237 | 0.0350111 | 3.35E-05 | 0.134237 | 0.0107 | 0.0151 | 0.4801 | 0.1849 |

Abbreviation: SNP, single nucleotide polymorphism; EA, Effect allele; OA, other allele; beta, estimate coefficient; se, standard error of coefficient estimate; eaf, effect allele frequency; ALS, amyotrophic lateral sclerosis.

**Table S3. Detailed information on the instrumental variables (IVs) for HSV keratitis, keratoconjunctivitis, and ALS in the forward MR analysis.**

| **SNP** | **EA** | **OA** | **Exposure (HSV keratitis and keratoconjunctivitis)** | | | | **Outcome (ALS)** | | | |
| --- | --- | --- | --- | --- | --- | --- | --- | --- | --- | --- |
|  |  |  | **beta** | **se** | **P** | **eaf** | **beta** | **se** | **P** | **eaf** |
| rs10206069 | C | T | 0.246476 | 0.0597983 | 3.76E-05 | 0.124321 | -0.004 | 0.0187 | 0.8299 | 0.106 |
| rs10509774 | T | C | -0.301088 | 0.072338 | 3.15E-05 | 0.108052 | -0.0177 | 0.0216 | 0.4141 | 0.0747 |
| rs114928340 | T | C | 0.874225 | 0.210733 | 3.35E-05 | 0.00531349 | -0.0345 | 0.0412 | 0.4022 | 0.0193 |
| rs11578616 | C | A | -0.209377 | 0.0499801 | 2.80E-05 | 0.246715 | 0.0188 | 0.0134 | 0.1619 | 0.2192 |
| rs117470581 | T | C | -1.01075 | 0.215456 | 2.72E-06 | 0.0174369 | -0.0305 | 0.039 | 0.4348 | 0.0269 |
| rs117511082 | A | G | 0.537195 | 0.122426 | 1.14E-05 | 0.0212076 | -0.0169 | 0.0435 | 0.6979 | 0.0214 |
| rs117837757 | A | G | 0.541164 | 0.125468 | 1.61E-05 | 0.0195008 | 0.0824 | 0.0427 | 0.0538 | 0.0186 |
| rs11802122 | T | G | -0.226915 | 0.0556427 | 4.54E-05 | 0.18901 | -0.0039 | 0.0157 | 0.8045 | 0.1547 |
| rs12365133 | G | A | -0.172988 | 0.0415895 | 3.19E-05 | 0.512738 | -6.00E-04 | 0.0112 | 0.9568 | 0.4891 |
| rs12637537 | T | C | -0.246131 | 0.0562553 | 1.21E-05 | 0.188742 | -0.0074 | 0.0164 | 0.6521 | 0.1688 |
| rs1337208 | T | C | -0.18185 | 0.0435662 | 2.99E-05 | 0.678506 | -0.0119 | 0.0118 | 0.3103 | 0.6623 |
| rs1368965 | A | C | -0.183839 | 0.0437667 | 2.66E-05 | 0.682145 | -0.0043 | 0.0117 | 0.715 | 0.6539 |
| rs138165380 | A | G | -0.513844 | 0.116789 | 1.08E-05 | 0.045831 | -0.0321 | 0.0302 | 0.2887 | 0.0366 |
| rs1383818 | C | T | 0.510791 | 0.111129 | 4.30E-06 | 0.950773 | -0.0115 | 0.0334 | 0.7312 | 0.9652 |
| rs1385827 | A | G | 0.629083 | 0.147995 | 2.13E-05 | 0.0130467 | -0.0523 | 0.0597 | 0.3808 | 0.0118 |
| rs141051150 | G | A | -0.973416 | 0.233664 | 3.10E-05 | 0.0146707 | -0.0116 | 0.0595 | 0.846 | 0.0115 |
| rs145672563 | A | G | 1.57847 | 0.356027 | 9.27E-06 | 0.0012221 | -0.2179 | 0.0907 | 0.01623 | 0.0049 |
| rs150808839 | G | A | 1.54819 | 0.344595 | 7.03E-06 | 0.00125572 | -0.0494 | 0.0529 | 0.3505 | 0.0103 |
| rs17793601 | C | T | 0.178791 | 0.0425916 | 2.70E-05 | 0.362065 | -0.0159 | 0.0133 | 0.2328 | 0.3597 |
| rs17798550 | T | C | 0.208063 | 0.0509829 | 4.48E-05 | 0.18742 | 0.0069 | 0.013 | 0.5929 | 0.2467 |
| rs185510182 | T | C | 1.02844 | 0.246837 | 3.09E-05 | 0.00371671 | 0.0134 | 0.0568 | 0.8137 | 0.0114 |
| rs1964218 | A | G | 0.185228 | 0.0456643 | 4.99E-05 | 0.274547 | -0.0165 | 0.0163 | 0.3092 | 0.2381 |
| rs2087264 | G | A | -0.185177 | 0.0455103 | 4.72E-05 | 0.320711 | 0.0156 | 0.0124 | 0.209 | 0.291 |
| rs2168819 | A | G | 0.181539 | 0.0445467 | 4.60E-05 | 0.294615 | -0.0165 | 0.0121 | 0.1715 | 0.299 |
| rs2293688 | G | C | 0.17437 | 0.0429243 | 4.86E-05 | 0.342742 | 0.0116 | 0.0117 | 0.3202 | 0.3421 |
| rs260591 | G | A | 0.221361 | 0.048622 | 5.30E-06 | 0.728532 | -0.0016 | 0.012 | 0.891 | 0.6726 |
| rs4757388 | G | A | 0.219127 | 0.0474343 | 3.84E-06 | 0.715308 | 0.0066 | 0.0115 | 0.5689 | 0.5853 |
| rs55848335 | T | A | -0.198422 | 0.0479519 | 3.50E-05 | 0.27788 | 0.0251 | 0.0126 | 0.04711 | 0.3253 |
| rs56757613 | T | G | -0.548664 | 0.129307 | 2.20E-05 | 0.0365614 | 0.0805 | 0.0287 | 0.004953 | 0.0392 |
| rs57817160 | A | G | -0.198995 | 0.0437142 | 5.31E-06 | 0.367568 | -0.0072 | 0.0116 | 0.5346 | 0.3449 |
| rs62115085 | T | C | 0.192864 | 0.0440692 | 1.21E-05 | 0.302702 | -0.0185 | 0.013 | 0.1547 | 0.3318 |
| rs62459582 | A | G | -0.6382 | 0.154983 | 3.82E-05 | 0.0269768 | -0.0306 | 0.0469 | 0.5141 | 0.0196 |
| rs6600441 | A | G | 0.457919 | 0.0985759 | 3.40E-06 | 0.939639 | 0.0368 | 0.0289 | 0.2038 | 0.9449 |
| rs71642840 | T | G | 0.760404 | 0.185623 | 4.19E-05 | 0.00784587 | 0.0522 | 0.0359 | 0.1452 | 0.0263 |
| rs72648746 | A | G | -0.296099 | 0.0666099 | 8.78E-06 | 0.128113 | -0.0207 | 0.02 | 0.302 | 0.0864 |
| rs73053679 | T | C | -0.885583 | 0.211453 | 2.81E-05 | 0.0167253 | 0.0659 | 0.0401 | 0.1008 | 0.0195 |
| rs73058103 | C | G | 0.4565 | 0.107007 | 1.99E-05 | 0.0294156 | -0.0011 | 0.0256 | 0.9655 | 0.0524 |
| rs74687283 | A | G | -0.374566 | 0.0880724 | 2.11E-05 | 0.0738459 | -0.0186 | 0.0196 | 0.343 | 0.0961 |
| rs74775034 | A | G | -0.632694 | 0.14495 | 1.27E-05 | 0.0312094 | 0.014 | 0.0312 | 0.6529 | 0.0416 |
| rs74828319 | G | A | -0.649801 | 0.150547 | 1.59E-05 | 0.0298953 | 0.0191 | 0.0376 | 0.6121 | 0.0266 |
| rs75088484 | G | A | -0.507398 | 0.118794 | 1.94E-05 | 0.043309 | -3.00E-04 | 0.0379 | 0.9928 | 0.0495 |
| rs75427405 | T | C | -0.427191 | 0.0968588 | 1.03E-05 | 0.0618307 | 0.0294 | 0.021 | 0.1609 | 0.0741 |
| rs75896254 | T | C | 0.431025 | 0.101271 | 2.08E-05 | 0.0327563 | -0.0153 | 0.0309 | 0.6207 | 0.0444 |
| rs76007602 | A | C | 0.408793 | 0.0984795 | 3.31E-05 | 0.0365135 | -0.0276 | 0.0325 | 0.3948 | 0.0395 |
| rs7604094 | G | T | 0.187966 | 0.0437206 | 1.71E-05 | 0.544308 | 0.0014 | 0.0124 | 0.9107 | 0.5245 |
| rs76188912 | T | C | 0.479151 | 0.117202 | 4.35E-05 | 0.0235198 | -0.008 | 0.0247 | 0.7467 | 0.0589 |
| rs76284770 | G | A | 0.489443 | 0.115847 | 2.39E-05 | 0.0240606 | 0.0021 | 0.0262 | 0.9373 | 0.0468 |
| rs771974 | A | G | -0.291739 | 0.0697985 | 2.92E-05 | 0.919283 | 0.0203 | 0.0195 | 0.2986 | 0.9099 |
| rs78742138 | C | T | 0.434161 | 0.0900348 | 1.42E-06 | 0.0417909 | 0.0144 | 0.0365 | 0.694 | 0.0257 |
| rs79284209 | C | T | 0.336133 | 0.075248 | 7.93E-06 | 0.0665494 | 0.0232 | 0.0208 | 0.2655 | 0.0792 |
| rs80296482 | T | C | 0.59452 | 0.145565 | 4.42E-05 | 0.013963 | -0.0382 | 0.0332 | 0.2502 | 0.0276 |
| rs8074012 | C | T | -1.51672 | 0.369188 | 3.99E-05 | 0.00824674 | 0.0384 | 0.0369 | 0.2978 | 0.024 |
| rs9309089 | A | G | 0.19779 | 0.0468024 | 2.38E-05 | 0.244147 | -0.0028 | 0.0137 | 0.84 | 0.2011 |
| rs9394200 | C | T | 0.71613 | 0.171892 | 3.10E-05 | 0.976823 | 0.0234 | 0.0325 | 0.4713 | 0.9646 |
| rs9612335 | A | C | -0.170821 | 0.0416599 | 4.12E-05 | 0.573129 | -0.0434 | 0.0169 | 0.01034 | 0.5821 |

Abbreviation: SNP, single nucleotide polymorphism; EA, Effect allele; OA, other allele; beta, estimate coefficient; se, standard error of coefficient estimate; eaf, effect allele frequency; ALS, amyotrophic lateral sclerosis.

**Table S4. Detailed information on the instrumental variables (IVs) for Anogenital HSV infection and ALS in the forward MR analysis.**

| **SNP** | **EA** | **OA** | **Exposure (Anogenital HSV infection)** | | | | **Outcome (ALS)** | | | |
| --- | --- | --- | --- | --- | --- | --- | --- | --- | --- | --- |
|  |  |  | **beta** | **se** | **P** | **eaf** | **beta** | **se** | **P** | **eaf** |
| rs10166518 | T | C | 0.142296 | 0.034824 | 4.39E-05 | 0.629271 | -0.0044 | 0.0117 | 0.7069 | 0.6618 |
| rs10484922 | T | C | -0.253258 | 0.0522413 | 1.25E-06 | 0.131991 | -0.0154 | 0.0197 | 0.4345 | 0.0882 |
| rs11067218 | C | T | 0.136201 | 0.0335077 | 4.81E-05 | 0.43972 | -0.0038 | 0.0123 | 0.7574 | 0.4476 |
| rs111488175 | A | T | 0.278294 | 0.06741 | 3.65E-05 | 0.0555181 | 0.0436 | 0.0312 | 0.1623 | 0.0367 |
| rs11237627 | A | G | 0.21312 | 0.0467356 | 5.11E-06 | 0.135887 | 0.003 | 0.0252 | 0.9044 | 0.0584 |
| rs114992997 | A | G | 0.50891 | 0.125432 | 4.97E-05 | 0.0135011 | -0.0144 | 0.0442 | 0.7437 | 0.0245 |
| rs115254282 | T | C | 0.495873 | 0.117692 | 2.52E-05 | 0.0153333 | 0.1016 | 0.0475 | 0.03255 | 0.0194 |
| rs1156233 | T | C | 0.159376 | 0.0378993 | 2.61E-05 | 0.23986 | 0.001 | 0.0129 | 0.9399 | 0.2472 |
| rs116304193 | A | C | -1.10757 | 0.250348 | 9.68E-06 | 0.00883833 | -0.1057 | 0.0427 | 0.01328 | 0.0218 |
| rs11725412 | G | A | 0.277962 | 0.0657155 | 2.34E-05 | 0.918878 | -0.0475 | 0.0237 | 0.0451 | 0.9342 |
| rs117318797 | T | C | 0.53118 | 0.129971 | 4.37E-05 | 0.0126742 | 0.0217 | 0.0475 | 0.6476 | 0.021 |
| rs117825741 | C | G | 0.621406 | 0.146131 | 2.11E-05 | 0.00932659 | 0.0229 | 0.0371 | 0.5362 | 0.0271 |
| rs118081726 | T | G | -0.44554 | 0.102922 | 1.50E-05 | 0.0349385 | -0.0185 | 0.0429 | 0.6656 | 0.0196 |
| rs118086501 | T | C | -0.835502 | 0.197558 | 2.35E-05 | 0.0117091 | 0.0718 | 0.0764 | 0.3473 | 0.0062 |
| rs1267858 | T | C | -0.251356 | 0.0618124 | 4.77E-05 | 0.0918962 | -0.0127 | 0.0208 | 0.5404 | 0.0748 |
| rs12967536 | A | G | 0.209317 | 0.0501962 | 3.05E-05 | 0.113275 | -0.0155 | 0.0198 | 0.4326 | 0.1007 |
| rs12987038 | T | C | -0.162099 | 0.037807 | 1.81E-05 | 0.760023 | 0.0108 | 0.0134 | 0.4213 | 0.7721 |
| rs13025262 | G | T | -0.23377 | 0.0558423 | 2.84E-05 | 0.112847 | 0.0242 | 0.0164 | 0.14 | 0.1649 |
| rs13083031 | T | C | 0.233726 | 0.0540184 | 1.51E-05 | 0.0919896 | -0.0128 | 0.017 | 0.4504 | 0.1295 |
| rs13232385 | G | C | 0.7211 | 0.154329 | 2.98E-06 | 0.00750701 | 0.0401 | 0.0359 | 0.2632 | 0.0262 |
| rs13426424 | A | G | 0.162597 | 0.0400077 | 4.82E-05 | 0.201545 | -0.0167 | 0.0136 | 0.2184 | 0.2173 |
| rs138004419 | A | G | -0.574435 | 0.140409 | 4.29E-05 | 0.0205995 | 0.0864 | 0.0685 | 0.2071 | 0.0071 |
| rs138265519 | C | A | -0.750015 | 0.183311 | 4.29E-05 | 0.0138049 | -0.0115 | 0.0459 | 0.8029 | 0.0243 |
| rs144855048 | A | G | -0.398157 | 0.0954692 | 3.04E-05 | 0.0407101 | -0.0325 | 0.0291 | 0.264 | 0.0426 |
| rs145588635 | T | C | -0.342158 | 0.0753184 | 5.55E-06 | 0.0634442 | 0.0227 | 0.0702 | 0.7461 | 0.0096 |
| rs16926436 | A | G | 0.200608 | 0.0493851 | 4.86E-05 | 0.115306 | -0.004 | 0.0172 | 0.8177 | 0.1292 |
| rs17019771 | G | A | -0.17141 | 0.0396907 | 1.57E-05 | 0.250384 | 0.0244 | 0.0138 | 0.07802 | 0.209 |
| rs17337014 | G | A | 0.267967 | 0.0611824 | 1.19E-05 | 0.0687803 | 0.0024 | 0.0191 | 0.9011 | 0.0945 |
| rs17466209 | T | A | -0.171951 | 0.041892 | 4.05E-05 | 0.212366 | -0.0065 | 0.0129 | 0.6165 | 0.2662 |
| rs17477055 | G | T | -0.334808 | 0.080114 | 2.93E-05 | 0.0550596 | 0.0188 | 0.0197 | 0.3397 | 0.0857 |
| rs17625475 | T | G | 0.210088 | 0.0495315 | 2.22E-05 | 0.115335 | -0.0072 | 0.02 | 0.7176 | 0.1162 |
| rs2070305 | G | T | -0.135829 | 0.0333162 | 4.56E-05 | 0.471163 | 0.0059 | 0.0112 | 0.5994 | 0.4329 |
| rs2305191 | T | C | -0.14391 | 0.0338323 | 2.10E-05 | 0.427082 | -0.0224 | 0.0113 | 0.04669 | 0.4042 |
| rs2437091 | T | C | 0.149667 | 0.0350571 | 1.96E-05 | 0.636051 | -0.0083 | 0.0126 | 0.5105 | 0.7411 |
| rs2604850 | C | T | 0.363135 | 0.0894442 | 4.91E-05 | 0.953356 | -0.0648 | 0.031 | 0.03688 | 0.9573 |
| rs2709114 | G | A | 0.225414 | 0.0541521 | 3.15E-05 | 0.881235 | 0.0432 | 0.0152 | 0.004363 | 0.8445 |
| rs28456023 | C | T | 0.200215 | 0.0491442 | 4.62E-05 | 0.116407 | -0.0063 | 0.0155 | 0.6852 | 0.1772 |
| rs36073836 | T | C | 0.188579 | 0.0462836 | 4.61E-05 | 0.136135 | -0.0244 | 0.0156 | 0.1174 | 0.1513 |
| rs3732148 | T | C | -0.333293 | 0.081148 | 4.00E-05 | 0.0554307 | 0.0173 | 0.0244 | 0.4782 | 0.0732 |
| rs4660006 | G | A | -0.20189 | 0.0394385 | 3.07E-07 | 0.791535 | -0.0204 | 0.0133 | 0.1251 | 0.7741 |
| rs4753106 | A | G | -0.140449 | 0.0333129 | 2.49E-05 | 0.557745 | 0.0074 | 0.0114 | 0.5139 | 0.476 |
| rs4817294 | A | G | -0.211737 | 0.0518317 | 4.41E-05 | 0.132121 | -0.014 | 0.0175 | 0.4222 | 0.1164 |
| rs60168603 | G | A | 0.183732 | 0.0439701 | 2.93E-05 | 0.155565 | -0.009 | 0.0156 | 0.5633 | 0.1565 |
| rs6489191 | G | A | 0.151436 | 0.0362122 | 2.89E-05 | 0.668419 | -0.0102 | 0.0115 | 0.3748 | 0.5985 |
| rs72972620 | G | A | -0.209017 | 0.0465713 | 7.19E-06 | 0.164983 | 0.0033 | 0.0156 | 0.8351 | 0.1423 |
| rs74298643 | T | C | 0.183733 | 0.0442457 | 3.29E-05 | 0.155757 | 0.0039 | 0.0165 | 0.8138 | 0.154 |
| rs74412266 | A | C | 0.560369 | 0.135685 | 3.63E-05 | 0.0104417 | 0.0329 | 0.0282 | 0.2446 | 0.0398 |
| rs7524977 | C | T | -0.941749 | 0.225889 | 3.06E-05 | 0.00989665 | 0.0394 | 0.0526 | 0.4542 | 0.0152 |
| rs77189104 | C | T | 0.33272 | 0.080479 | 3.56E-05 | 0.0384544 | -0.1101 | 0.0587 | 0.06054 | 0.0236 |
| rs77602229 | C | T | -0.377391 | 0.0918487 | 3.98E-05 | 0.0416995 | 0.0285 | 0.0522 | 0.5848 | 0.025 |
| rs79413341 | C | A | -0.290228 | 0.0690638 | 2.64E-05 | 0.0731826 | 0.0601 | 0.0289 | 0.03736 | 0.0482 |
| rs7941503 | T | C | 0.140699 | 0.0343568 | 4.22E-05 | 0.366963 | -0.0048 | 0.0117 | 0.6808 | 0.3806 |
| rs903217 | A | T | 0.143368 | 0.034701 | 3.60E-05 | 0.329653 | 0.0138 | 0.0113 | 0.222 | 0.3851 |
| rs9508320 | A | G | 0.512668 | 0.125263 | 4.26E-05 | 0.012722 | 0.093 | 0.0439 | 0.03427 | 0.0202 |
| rs9566497 | C | T | -0.250344 | 0.0613657 | 4.51E-05 | 0.0922213 | -0.021 | 0.0169 | 0.2163 | 0.1197 |
| rs962115 | C | A | -0.14584 | 0.0356032 | 4.20E-05 | 0.704313 | -0.0098 | 0.0136 | 0.4717 | 0.7795 |

Abbreviation: SNP, single nucleotide polymorphism; EA, Effect allele; OA, other allele; beta, estimate coefficient; se, standard error of coefficient estimate; eaf, effect allele frequency; ALS, amyotrophic lateral sclerosis.

**Table S5. Detailed information on the instrumental variables (IVs) for VZV IgG and ALS in the forward MR analysis.**

| **SNP** | **EA** | **OA** | **Exposure (****VZV IgG)** | | | | **Outcome (ALS)** | | | |
| --- | --- | --- | --- | --- | --- | --- | --- | --- | --- | --- |
|  |  |  | **beta** | **se** | **P** | **eaf** | **beta** | **se** | **P** | **eaf** |
| rs10185554 | A | G | 0.560054 | 0.132806 | 2.47E-05 | 0.0275645 | 0.0401 | 0.0314 | 0.2024 | 0.0345 |
| rs10438342 | A | G | 0.199867 | 0.0451572 | 9.60E-06 | 0.343837 | -0.0069 | 0.0115 | 0.5499 | 0.362 |
| rs11205012 | C | T | -0.191538 | 0.0461511 | 3.32E-05 | 0.322259 | 0.013 | 0.0123 | 0.291 | 0.339 |
| rs116920297 | A | G | -0.646066 | 0.152773 | 2.35E-05 | 0.0205235 | 0.0357 | 0.0416 | 0.3911 | 0.0223 |
| rs117250441 | C | T | -0.515085 | 0.114494 | 6.83E-06 | 0.0354322 | -0.0093 | 0.0339 | 0.784 | 0.0312 |
| rs117300637 | G | C | -0.964974 | 0.234649 | 3.92E-05 | 0.00916638 | -0.0868 | 0.0841 | 0.3016 | 0.0109 |
| rs117403751 | A | T | -0.649889 | 0.149117 | 1.31E-05 | 0.0217187 | -0.0375 | 0.0409 | 0.36 | 0.0293 |
| rs11933473 | C | G | -0.497963 | 0.119153 | 2.93E-05 | 0.0338808 | -0.0334 | 0.0308 | 0.2773 | 0.0339 |
| rs12297976 | A | G | -0.186735 | 0.0435647 | 1.82E-05 | 0.442559 | 0.0027 | 0.0121 | 0.8209 | 0.4422 |
| rs1283728 | A | G | -0.681653 | 0.165358 | 3.75E-05 | 0.017974 | 0.008 | 0.0439 | 0.8554 | 0.0179 |
| rs12975162 | T | C | 0.209493 | 0.0480065 | 1.28E-05 | 0.270651 | -0.0242 | 0.0133 | 0.06923 | 0.262 |
| rs13208342 | A | T | 0.191453 | 0.0446924 | 1.84E-05 | 0.36994 | 0.0135 | 0.0116 | 0.2423 | 0.3828 |
| rs13294227 | T | C | -0.768909 | 0.180629 | 2.07E-05 | 0.0148254 | 0.0095 | 0.0514 | 0.8532 | 0.0144 |
| rs13294894 | A | G | -1.19814 | 0.27836 | 1.68E-05 | 0.00659252 | -0.0129 | 0.0547 | 0.8132 | 0.0098 |
| rs13406838 | T | C | -0.249838 | 0.0602038 | 3.33E-05 | 0.14558 | 0.0037 | 0.016 | 0.8182 | 0.1547 |
| rs139359209 | G | C | -1.12859 | 0.237543 | 2.02E-06 | 0.00925181 | 4.00E-04 | 0.0492 | 0.9933 | 0.0135 |
| rs139406952 | C | T | -0.769174 | 0.188638 | 4.55E-05 | 0.0140341 | -0.0245 | 0.0371 | 0.5085 | 0.0257 |
| rs145339536 | A | G | -0.66461 | 0.144872 | 4.48E-06 | 0.0228928 | 0.0681 | 0.0346 | 0.04931 | 0.0287 |
| rs145749545 | T | G | -0.797598 | 0.168087 | 2.08E-06 | 0.0173788 | 0.0504 | 0.0479 | 0.2922 | 0.018 |
| rs146203692 | T | C | -1.15244 | 0.255109 | 6.26E-06 | 0.00787266 | -0.0485 | 0.0584 | 0.4063 | 0.0098 |
| rs146435600 | C | G | 0.894674 | 0.220034 | 4.78E-05 | 0.0100265 | 0.09 | 0.0577 | 0.119 | 0.0114 |
| rs147224930 | T | G | -1.23006 | 0.288633 | 2.03E-05 | 0.00628604 | 0.0033 | 0.0703 | 0.9621 | 0.0087 |
| rs1473752 | T | C | -0.324016 | 0.0791123 | 4.21E-05 | 0.919191 | 0.0436 | 0.0195 | 0.02514 | 0.9115 |
| rs150171569 | A | C | -0.732065 | 0.172681 | 2.24E-05 | 0.0158101 | -0.0758 | 0.0478 | 0.1128 | 0.0163 |
| rs150502985 | A | G | -1.12203 | 0.264798 | 2.26E-05 | 0.00726811 | -0.0413 | 0.0629 | 0.511 | 0.0103 |
| rs150943326 | A | G | 0.665102 | 0.151933 | 1.20E-05 | 0.0213938 | -0.0551 | 0.0418 | 0.1869 | 0.0255 |
| rs1556124 | A | G | 0.218473 | 0.0502278 | 1.36E-05 | 0.769548 | -0.0098 | 0.0152 | 0.5177 | 0.7778 |
| rs17816565 | G | A | -0.877737 | 0.213816 | 4.04E-05 | 0.0105251 | 0.0545 | 0.0515 | 0.2895 | 0.0134 |
| rs2025227 | A | C | 0.187011 | 0.0438479 | 2.00E-05 | 0.35945 | 0.015 | 0.0114 | 0.1904 | 0.3583 |
| rs2029970 | C | T | -0.190919 | 0.047066 | 4.98E-05 | 0.716376 | 0.0113 | 0.0125 | 0.3663 | 0.7196 |
| rs2100402 | G | A | -0.207468 | 0.0505043 | 3.99E-05 | 0.237224 | 0.0267 | 0.0131 | 0.0415 | 0.2414 |
| rs2495717 | G | A | -0.175087 | 0.0424319 | 3.69E-05 | 0.491757 | -0.0046 | 0.0116 | 0.6932 | 0.4997 |
| rs2949329 | T | C | 0.30935 | 0.0718159 | 1.65E-05 | 0.0950518 | -0.0035 | 0.0174 | 0.8423 | 0.1102 |
| rs34833393 | T | G | -0.183681 | 0.0431429 | 2.07E-05 | 0.451257 | 0.0112 | 0.012 | 0.3541 | 0.4488 |
| rs3807593 | G | T | -0.513958 | 0.121585 | 2.37E-05 | 0.0324181 | 0.0116 | 0.034 | 0.7338 | 0.0308 |
| rs4395772 | C | T | 0.327009 | 0.0742231 | 1.05E-05 | 0.904848 | 0.0091 | 0.0197 | 0.6425 | 0.8814 |
| rs445518 | T | C | 0.196513 | 0.0457973 | 1.78E-05 | 0.330203 | -0.014 | 0.012 | 0.2433 | 0.3271 |
| rs56272852 | G | T | -0.814504 | 0.194951 | 2.94E-05 | 0.0127065 | 0.0313 | 0.0506 | 0.537 | 0.0163 |
| rs6007320 | G | A | 0.566737 | 0.129961 | 1.30E-05 | 0.0288274 | 0.0318 | 0.0346 | 0.3581 | 0.0282 |
| rs6022713 | C | T | -0.179959 | 0.0436036 | 3.67E-05 | 0.407525 | 2.00E-04 | 0.0114 | 0.9844 | 0.4044 |
| rs61997108 | A | C | 0.213415 | 0.0519734 | 4.02E-05 | 0.219632 | -0.0064 | 0.0155 | 0.6785 | 0.2096 |
| rs62473135 | C | G | -0.325315 | 0.0703844 | 3.80E-06 | 0.102007 | -0.0306 | 0.0187 | 0.1015 | 0.1038 |
| rs67087083 | T | C | 0.237665 | 0.0585505 | 4.93E-05 | 0.161782 | 0.0276 | 0.0155 | 0.0744 | 0.1645 |
| rs699242 | A | G | -0.265112 | 0.0562388 | 2.43E-06 | 0.826903 | -0.0108 | 0.0142 | 0.4467 | 0.8024 |
| rs721294 | G | T | -0.392941 | 0.0863689 | 5.38E-06 | 0.0689469 | -0.0149 | 0.0233 | 0.5215 | 0.0714 |
| rs73222743 | A | G | -1.08029 | 0.255151 | 2.30E-05 | 0.00771014 | 0.027 | 0.0573 | 0.6375 | 0.0126 |
| rs74424451 | T | C | -0.648874 | 0.158517 | 4.25E-05 | 0.0186792 | -0.0027 | 0.0471 | 0.955 | 0.021 |
| rs75281625 | T | C | -0.519817 | 0.121021 | 1.74E-05 | 0.0315662 | 0.0574 | 0.035 | 0.1012 | 0.0343 |
| rs75618462 | A | G | -0.34071 | 0.0781147 | 1.29E-05 | 0.0798512 | -0.0097 | 0.0217 | 0.6538 | 0.0826 |
| rs75623848 | G | C | -0.691351 | 0.161623 | 1.89E-05 | 0.0184316 | 0.0506 | 0.0444 | 0.2538 | 0.0211 |
| rs75651344 | T | A | -0.480389 | 0.0953268 | 4.67E-07 | 0.054108 | 0.0072 | 0.0244 | 0.7688 | 0.0631 |
| rs75967781 | A | C | 0.341492 | 0.082237 | 3.29E-05 | 0.0751982 | 0.0018 | 0.0196 | 0.9278 | 0.0937 |
| rs76174378 | A | G | -0.455404 | 0.107996 | 2.48E-05 | 0.0413247 | 0.0522 | 0.03 | 0.08183 | 0.0387 |
| rs76940747 | T | C | -0.790173 | 0.187643 | 2.54E-05 | 0.0140417 | -0.0895 | 0.0492 | 0.06914 | 0.0146 |
| rs77177098 | G | A | -0.798164 | 0.194724 | 4.15E-05 | 0.0127674 | -0.0351 | 0.0432 | 0.4167 | 0.0177 |
| rs77591080 | A | G | -0.670402 | 0.143067 | 2.79E-06 | 0.0233559 | 0.0094 | 0.041 | 0.8181 | 0.0221 |
| rs78181716 | T | C | -0.665348 | 0.155727 | 1.93E-05 | 0.020014 | -0.0139 | 0.043 | 0.7465 | 0.0217 |
| rs78192503 | A | T | -0.213709 | 0.0465516 | 4.42E-06 | 0.336073 | -0.008 | 0.0132 | 0.5444 | 0.3385 |
| rs7900528 | T | G | -0.268627 | 0.0656013 | 4.22E-05 | 0.118985 | -0.0056 | 0.0173 | 0.7447 | 0.1248 |
| rs79095325 | A | G | -0.20215 | 0.0489799 | 3.67E-05 | 0.253009 | -0.002 | 0.0127 | 0.8756 | 0.2559 |
| rs80026625 | A | T | 0.6276 | 0.149479 | 2.69E-05 | 0.0217341 | 0.0544 | 0.0372 | 0.1433 | 0.0272 |
| rs8141472 | T | C | 0.186789 | 0.0457635 | 4.47E-05 | 0.370908 | -0.007 | 0.013 | 0.5917 | 0.3877 |
| rs9266639 | T | C | 0.196322 | 0.0466551 | 2.58E-05 | 0.286909 | 0.0331 | 0.0124 | 0.007695 | 0.2769 |
| rs9295829 | G | A | -0.242872 | 0.0473848 | 2.97E-07 | 0.274356 | 0.0151 | 0.0125 | 0.2251 | 0.2752 |
| rs9525863 | A | G | -0.487728 | 0.111109 | 1.14E-05 | 0.0390384 | 0.0104 | 0.0255 | 0.6822 | 0.047 |

Abbreviation: SNP, single nucleotide polymorphism; EA, Effect allele; OA, other allele; beta, estimate coefficient; se, standard error of coefficient estimate; eaf, effect allele frequency; ALS, amyotrophic lateral sclerosis.

**Table S6. Detailed information on the EBV and ALS instrumental variables (IVs) in the forward MR analysis.**

| **SNP** | **EA** | **OA** | **Exposure (EBV)** | | | | **Outcome (ALS)** | | | |
| --- | --- | --- | --- | --- | --- | --- | --- | --- | --- | --- |
|  |  |  | **beta** | **se** | **P** | **eaf** | **beta** | **se** | **P** | **eaf** |
| rs1054765 | A | C | -0.160851 | 0.0385281 | 2.98E-05 | 0.839535 | -0.0065 | 0.014 | 0.6423 | 0.8015 |
| rs1417573 | A | T | 0.175901 | 0.0356565 | 8.09E-07 | 0.197221 | -2.00E-04 | 0.0139 | 0.9872 | 0.1927 |
| rs17313034 | C | T | -0.146924 | 0.0339207 | 1.48E-05 | 0.267227 | -0.001 | 0.0121 | 0.9349 | 0.3084 |
| rs17426492 | A | G | 0.345936 | 0.0852659 | 4.97E-05 | 0.0241986 | -0.0492 | 0.0299 | 0.0994 | 0.035 |
| rs61823776 | T | A | -0.199634 | 0.0488296 | 4.34E-05 | 0.117262 | 0.0234 | 0.0194 | 0.2262 | 0.1306 |
| rs9943200 | C | T | -0.139796 | 0.0332357 | 2.60E-05 | 0.287166 | 0.0161 | 0.0119 | 0.1757 | 0.3335 |

Abbreviation: SNP, single nucleotide polymorphism; EA, Effect allele; OA, other allele; beta, estimate coefficient; se, standard error of coefficient estimate; eaf, effect allele frequency; ALS, amyotrophic lateral sclerosis.

**Table S7. Detailed information on the CMV IgG and ALS instrumental variables (IVs) in the forward MR analysis.**

| **SNP** | **EA** | **OA** | **Exposure (CMV IgG)** | | | | **Outcome (ALS)** | | | |
| --- | --- | --- | --- | --- | --- | --- | --- | --- | --- | --- |
|  |  |  | **beta** | **se** | **P** | **eaf** | **beta** | **se** | **P** | **eaf** |
| rs10754470 | G | A | 0.100463 | 0.0226194 | 9.18E-06 | 0.43456 | 0.0101 | 0.0114 | 0.3743 | 0.4471 |
| rs13026783 | C | G | 0.344527 | 0.0845332 | 4.68E-05 | 0.019227 | -0.0462 | 0.0455 | 0.3101 | 0.0207 |
| rs146990284 | A | G | 0.416181 | 0.0855153 | 1.18E-06 | 0.016825 | -0.0298 | 0.0493 | 0.5464 | 0.0151 |
| rs17030379 | A | G | 0.200724 | 0.0447383 | 7.45E-06 | 0.069338 | -0.0098 | 0.023 | 0.6696 | 0.0668 |
| rs3739077 | T | C | 0.0993517 | 0.0227082 | 1.25E-05 | 0.4198 | -0.0029 | 0.0114 | 0.7978 | 0.421 |
| rs55932172 | G | A | 0.275684 | 0.063435 | 1.42E-05 | 0.03163 | -0.0767 | 0.038 | 0.04363 | 0.0261 |
| rs78798345 | C | T | -0.470242 | 0.104973 | 7.69E-06 | 0.011526 | 0.0038 | 0.0521 | 0.9414 | 0.0132 |

Abbreviation: SNP, single nucleotide polymorphism; EA, Effect allele; OA, other allele; beta, estimate coefficient; se, standard error of coefficient estimate; eaf, effect allele frequency; ALS, amyotrophic lateral sclerosis.

**Table S8. Detailed information on the instrumental variables (IVs) for HHV-6 IgG and ALS in the forward MR analysis.**

| **SNP** | **EA** | **OA** | **Exposure (HHV-6 IgG)** | | | | **Outcome (ALS)** | | | |
| --- | --- | --- | --- | --- | --- | --- | --- | --- | --- | --- |
|  |  |  | **beta** | **se** | **P** | **eaf** | **beta** | **se** | **P** | **eaf** |
| rs10193761 | T | C | 0.410777 | 0.101018 | 4.78E-05 | NA | 0.0081 | 0.0188 | 0.6682 | 0.9049 |
| rs142041262 | T | G | -0.947028 | 0.20057 | 2.34E-06 | NA | -0.0226 | 0.0433 | 0.601 | 0.0204 |
| rs2308941 | A | G | -0.867024 | 0.202538 | 1.86E-05 | NA | 0.0229 | 0.0527 | 0.6638 | 0.0163 |
| rs2799671 | T | C | -0.882115 | 0.185737 | 2.04E-06 | NA | 0.0381 | 0.0332 | 0.2506 | 0.0321 |
| rs75219682 | T | C | -0.399977 | 0.0973627 | 3.99E-05 | NA | 0.0113 | 0.0214 | 0.5988 | 0.0842 |

Abbreviation: SNP, single nucleotide polymorphism; EA, Effect allele; OA, other allele; beta, estimate coefficient; se, standard error of coefficient estimate; eaf, effect allele frequency; ALS, amyotrophic lateral sclerosis.

**Table S9. Detailed information on the instrumental variables (IVs) for HHV-7 IgG and ALS in the forward MR analysis.**

| **SNP** | **EA** | **OA** | **Exposure (HHV-7 IgG)** | | | | **Outcome (ALS)** | | | |
| --- | --- | --- | --- | --- | --- | --- | --- | --- | --- | --- |
|  |  |  | **beta** | **se** | **P** | **eaf** | **beta** | **se** | **P** | **eaf** |
| rs116323441 | T | C | -1.19918 | 0.279689 | 1.81E-05 | NA | -0.0163 | 0.0465 | 0.727 | 0.0219 |
| rs12406839 | A | C | -0.806712 | 0.191863 | 2.62E-05 | NA | -0.014 | 0.0311 | 0.6529 | 0.0368 |
| rs2011274 | C | T | 0.495288 | 0.112867 | 1.14E-05 | NA | 0.0141 | 0.0176 | 0.4237 | 0.8802 |
| rs74136419 | T | G | -0.558768 | 0.132021 | 2.31E-05 | NA | 0.0146 | 0.0198 | 0.4603 | 0.1038 |
| rs75180372 | A | G | -0.785342 | 0.176136 | 8.24E-06 | NA | 0.0492 | 0.0281 | 0.0802 | 0.0463 |
| rs75645745 | T | G | -0.743656 | 0.183255 | 4.95E-05 | NA | -0.0234 | 0.0277 | 0.3975 | 0.0467 |

Abbreviation: SNP, single nucleotide polymorphism; EA, Effect allele; OA, other allele; beta, estimate coefficient; se, standard error of coefficient estimate; eaf, effect allele frequency; ALS, amyotrophic lateral sclerosis.

**Table S10. Detailed information on the instrumental variables (IVs) for ALS and HSV infections in the reverse MR analysis.**

| **SNP** | **EA** | **OA** | **Exposure (ALS)** | | | | **Outcome (HSV infections)** | | | |
| --- | --- | --- | --- | --- | --- | --- | --- | --- | --- | --- |
|  |  |  | **beta** | **se** | **P** | **eaf** | **beta** | **se** | **P** | **eaf** |
| rs111629730 | T | C | 0.1987 | 0.0475 | 2.87E-05 | 0.0153 | -0.0665251 | 0.0899181 | 0.459396458 | 0.0187903 |
| rs1214598 | A | G | -0.0555 | 0.0117 | 2.10E-06 | 0.371 | -0.0709856 | 0.0285221 | 0.012817761 | 0.247197 |
| rs139248895 | T | G | -0.1898 | 0.0463 | 4.14E-05 | 0.0162 | 0.134137 | 0.231581 | 0.562438835 | 0.00301107 |
| rs17524886 | T | C | -0.0491 | 0.011 | 8.06E-06 | 0.5154 | -0.0269416 | 0.0245701 | 0.272851384 | 0.475317 |
| rs2123331 | A | G | -0.0489 | 0.0115 | 2.12E-05 | 0.6409 | 0.0070571 | 0.0275458 | 0.797800164 | 0.725902 |
| rs2354982 | T | C | 0.0823 | 0.0193 | 2.01E-05 | 0.8988 | 0.0790125 | 0.0330774 | 0.016907387 | 0.83445 |
| rs2744680 | A | G | -0.0667 | 0.0149 | 7.59E-06 | 0.8343 | -0.0878679 | 0.0301965 | 0.003615787 | 0.803079 |
| rs4669231 | A | G | 0.136 | 0.033 | 3.77E-05 | 0.0414 | -0.0673593 | 0.0658974 | 0.306693592 | 0.0358933 |
| rs76553808 | T | C | -0.1002 | 0.0243 | 3.73E-05 | 0.0548 | 0.0944678 | 0.0672525 | 0.16011841 | 0.0343774 |

Abbreviation: SNP, single nucleotide polymorphism; EA, Effect allele; OA, other allele; beta, estimate coefficient; se, standard error of coefficient estimate; eaf, effect allele frequency; ALS, amyotrophic lateral sclerosis.

**Table S11. Detailed information on the instrumental variables (IVs) for ALS and HSV keratitis and keratoconjunctivitis in the reverse MR analysis.**

| **SNP** | **EA** | **OA** | **Exposure (ALS)** | | | | **Outcome (HSV keratitis and keratoconjunctivitis)** | | | |
| --- | --- | --- | --- | --- | --- | --- | --- | --- | --- | --- |
|  |  |  | **beta** | **se** | **P** | **eaf** | **beta** | **se** | **P** | **eaf** |
| rs111629730 | T | C | 0.1987 | 0.0475 | 2.87E-05 | 0.0153 | -0.219124 | 0.154253 | 0.155447 | 0.018808 |
| rs1214598 | A | G | -0.0555 | 0.0117 | 2.10E-06 | 0.371 | -0.0129303 | 0.0487963 | 0.791021 | 0.247296 |
| rs139248895 | T | G | -0.1898 | 0.0463 | 4.14E-05 | 0.0162 | -0.105108 | 0.394574 | 0.789943 | 0.0030135 |
| rs17524886 | T | C | -0.0491 | 0.011 | 8.06E-06 | 0.5154 | 0.0602721 | 0.0420273 | 0.151539 | 0.475387 |
| rs2123331 | A | G | -0.0489 | 0.0115 | 2.12E-05 | 0.6409 | 0.0183671 | 0.0471139 | 0.696651 | 0.726085 |
| rs2354982 | T | C | 0.0823 | 0.0193 | 2.01E-05 | 0.8988 | 0.0851364 | 0.0567782 | 0.133755 | 0.834369 |
| rs2744680 | A | G | -0.0667 | 0.0149 | 7.59E-06 | 0.8343 | -0.0196252 | 0.0527121 | 0.709663 | 0.802955 |
| rs4669231 | A | G | 0.136 | 0.033 | 3.77E-05 | 0.0414 | -0.165722 | 0.113337 | 0.143686 | 0.0359015 |
| rs76553808 | T | C | -0.1002 | 0.0243 | 3.73E-05 | 0.0548 | 0.0768128 | 0.114741 | 0.50321 | 0.0344209 |

Abbreviation: SNP, single nucleotide polymorphism; EA, Effect allele; OA, other allele; beta, estimate coefficient; se, standard error of coefficient estimate; eaf, effect allele frequency; ALS, amyotrophic lateral sclerosis.

**Table S12. Detailed information on the instrumental variables (IVs) for ALS and anogenital HSV infection in the reverse MR analysis.**

| **SNP** | **EA** | **OA** | **Exposure (ALS)** | | | | **Outcome (Anogenital HSV infection)** | | | |
| --- | --- | --- | --- | --- | --- | --- | --- | --- | --- | --- |
|  |  |  | **beta** | **se** | **P** | **eaf** | **beta** | **se** | **P** | **eaf** |
| rs111629730 | T | C | 0.1987 | 0.0475 | 2.87E-05 | 0.0153 | 0.185562 | 0.122901 | 0.131083 | 0.0187853 |
| rs1214598 | A | G | -0.0555 | 0.0117 | 2.10E-06 | 0.371 | 0.000396464 | 0.038828 | 0.991853 | 0.247189 |
| rs139248895 | T | G | -0.1898 | 0.0463 | 4.14E-05 | 0.0162 | 0.105558 | 0.306341 | 0.730412 | 0.00300067 |
| rs2123331 | A | G | -0.0489 | 0.0115 | 2.12E-05 | 0.6409 | 0.0250713 | 0.0375951 | 0.50485 | 0.72593 |
| rs2354982 | T | C | 0.0823 | 0.0193 | 2.01E-05 | 0.8988 | 0.0361078 | 0.0450022 | 0.422347 | 0.834489 |
| rs2744680 | A | G | -0.0667 | 0.0149 | 7.59E-06 | 0.8343 | -0.0620778 | 0.0420209 | 0.139593 | 0.803014 |
| rs4669231 | A | G | 0.136 | 0.033 | 3.77E-05 | 0.0414 | -0.0674237 | 0.090319 | 0.455361 | 0.0358307 |
| rs76553808 | T | C | -0.1002 | 0.0243 | 3.73E-05 | 0.0548 | 0.133458 | 0.0916228 | 0.145225 | 0.0343748 |

Abbreviation: SNP, single nucleotide polymorphism; EA, Effect allele; OA, other allele; beta, estimate coefficient; se, standard error of coefficient estimate; eaf, effect allele frequency; ALS, amyotrophic lateral sclerosis.

**Table S13. Detailed information on the ALS and VZV IgG instrumental variables (IVs) in the reverse MR analysis.**

| **SNP** | **EA** | **OA** | **Exposure (ALS)** | | | | **Outcome (VZV IgG)** | | | |
| --- | --- | --- | --- | --- | --- | --- | --- | --- | --- | --- |
|  |  |  | **beta** | **se** | **P** | **eaf** | **beta** | **se** | **P** | **eaf** |
| rs111629730 | T | C | 0.1987 | 0.0475 | 2.87E-05 | 0.0153 | 0.053981 | 0.180652 | 0.765083 | 0.0136584 |
| rs1214598 | A | G | -0.0555 | 0.0117 | 2.10E-06 | 0.371 | 0.00959297 | 0.0436864 | 0.826193 | 0.380301 |
| rs139248895 | T | G | -0.1898 | 0.0463 | 4.14E-05 | 0.0162 | -0.253202 | 0.150123 | 0.0916732 | 0.0200596 |
| rs17524886 | T | C | -0.0491 | 0.011 | 8.06E-06 | 0.5154 | 0.00335711 | 0.0422053 | 0.936601 | 0.516714 |
| rs2123331 | A | G | -0.0489 | 0.0115 | 2.12E-05 | 0.6409 | 0.0206761 | 0.0445831 | 0.642816 | 0.639298 |
| rs2354982 | T | C | 0.0823 | 0.0193 | 2.01E-05 | 0.8988 | -0.0732957 | 0.0756127 | 0.332367 | 0.9100245 |
| rs2744680 | A | G | -0.0667 | 0.0149 | 7.59E-06 | 0.8343 | -0.00603575 | 0.0587771 | 0.91821 | 0.847761 |
| rs4653570 | A | T | 0.0741 | 0.0148 | 5.54E-07 | 0.8092 | -0.00323749 | 0.0562604 | 0.954111 | 0.819248 |
| rs4669231 | A | G | 0.136 | 0.033 | 3.77E-05 | 0.0414 | -0.079862 | 0.10768 | 0.458293 | 0.0399198 |
| rs76553808 | T | C | -0.1002 | 0.0243 | 3.73E-05 | 0.0548 | 0.165532 | 0.0927108 | 0.0741864 | 0.0555556 |

Abbreviation: SNP, single nucleotide polymorphism; EA, Effect allele; OA, other allele; beta, estimate coefficient; se, standard error of coefficient estimate; eaf, effect allele frequency; ALS, amyotrophic lateral sclerosis.

**Table S14. Detailed information on ALS and EBV instrumental variables (IVs) in the reverse MR analysis.**

| **SNP** | **EA** | **OA** | **Exposure (ALS)** | | | | **Outcome (EBV)** | | | |
| --- | --- | --- | --- | --- | --- | --- | --- | --- | --- | --- |
|  |  |  | **beta** | **se** | **P** | **eaf** | **beta** | **se** | **P** | **eaf** |
| rs111629730 | T | C | 0.1987 | 0.0475 | 2.87E-05 | 0.0153 | -0.00141413 | 0.109384 | 0.989685 | 0.0187665 |
| rs1214598 | A | G | -0.0555 | 0.0117 | 2.10E-06 | 0.371 | 0.0187766 | 0.034202 | 0.58301 | 0.247323 |
| rs139248895 | T | G | -0.1898 | 0.0463 | 4.14E-05 | 0.0162 | 0.222043 | 0.269542 | 0.410064 | 0.00301734 |
| rs17524886 | T | C | -0.0491 | 0.011 | 8.06E-06 | 0.5154 | -0.00240459 | 0.0296111 | 0.935278 | 0.47515 |
| rs2123331 | A | G | -0.0489 | 0.0115 | 2.12E-05 | 0.6409 | 0.0185626 | 0.0330686 | 0.574569 | 0.72593 |
| rs2354982 | T | C | 0.0823 | 0.0193 | 2.01E-05 | 0.8988 | -0.0540585 | 0.0397607 | 0.173958 | 0.834397 |
| rs2744680 | A | G | -0.0667 | 0.0149 | 7.59E-06 | 0.8343 | -0.0406242 | 0.0371299 | 0.273906 | 0.802986 |
| rs4669231 | A | G | 0.136 | 0.033 | 3.77E-05 | 0.0414 | 0.0705424 | 0.078625 | 0.369612 | 0.0359321 |
| rs76553808 | T | C | -0.1002 | 0.0243 | 3.73E-05 | 0.0548 | 0.0382654 | 0.0811275 | 0.637162 | 0.0344195 |

Abbreviation: SNP, single nucleotide polymorphism; EA, Effect allele; OA, other allele; beta, estimate coefficient; se, standard error of coefficient estimate; eaf, effect allele frequency; ALS, amyotrophic lateral sclerosis.

**Table S15. Detailed information on the ALS and CMV IgG instrumental variables (IVs) in the reverse MR analysis.**

| **SNP** | **EA** | **OA** | **Exposure (ALS)** | | | | **Outcome (CMV IgG)** | | | |
| --- | --- | --- | --- | --- | --- | --- | --- | --- | --- | --- |
|  |  |  | **beta** | **se** | **P** | **eaf** | **beta** | **se** | **P** | **eaf** |
| rs111629730 | T | C | 0.1987 | 0.0475 | 2.87E-05 | 0.0153 | -0.0783542 | 0.0841427 | 0.351746935 | 0.017593 |
| rs1214598 | A | G | -0.0555 | 0.0117 | 2.10E-06 | 0.371 | 0.0103738 | 0.0231215 | 0.65367358 | 0.37898 |
| rs139248895 | T | G | -0.1898 | 0.0463 | 4.14E-05 | 0.0162 | 0.0726738 | 0.0814551 | 0.372288638 | 0.020161 |
| rs17524886 | T | C | -0.0491 | 0.011 | 8.06E-06 | 0.5154 | -0.0184482 | 0.0224854 | 0.411958264 | 0.48962 |
| rs2123331 | A | G | -0.0489 | 0.0115 | 2.12E-05 | 0.6409 | 0.0106709 | 0.0234216 | 0.648677058 | 0.64715 |
| rs2354982 | T | C | 0.0823 | 0.0193 | 2.01E-05 | 0.8988 | 0.0156661 | 0.0387584 | 0.686066513 | 0.904842 |
| rs2744680 | A | G | -0.0667 | 0.0149 | 7.59E-06 | 0.8343 | -0.0270825 | 0.0317697 | 0.393957058 | 0.15319 |
| rs4669231 | A | G | 0.136 | 0.033 | 3.77E-05 | 0.0414 | -0.13996 | 0.0590837 | 0.017843834 | 0.041261 |

Abbreviation: SNP, single nucleotide polymorphism; EA, Effect allele; OA, other allele; beta, estimate coefficient; se, standard error of coefficient estimate; eaf, effect allele frequency; ALS, amyotrophic lateral sclerosis.

**Table S16. Detailed information on the instrumental variables (IVs) for ALS and HHV-6 IgG in the reverse MR analysis.**

| **SNP** | **EA** | **OA** | **Exposure (ALS)** | | | | **Outcome (HHV-6 IgG)** | | | |
| --- | --- | --- | --- | --- | --- | --- | --- | --- | --- | --- |
|  |  |  | **beta** | **se** | **P** | **eaf** | **beta** | **se** | **P** | **eaf** |
| rs111629730 | T | C | 0.1987 | 0.0475 | 2.87E-05 | 0.0153 | 0.0338317 | 0.231005 | 0.883563 | 0.0136584 |
| rs1214598 | A | G | -0.0555 | 0.0117 | 2.10E-06 | 0.371 | -0.0230955 | 0.0553624 | 0.676555 | 0.380301 |
| rs139248895 | T | G | -0.1898 | 0.0463 | 4.14E-05 | 0.0162 | 0.089508 | 0.188915 | 0.635642 | 0.0200596 |
| rs17524886 | T | C | -0.0491 | 0.011 | 8.06E-06 | 0.5154 | -0.0456599 | 0.0535434 | 0.39379 | 0.516714 |
| rs2123331 | A | G | -0.0489 | 0.0115 | 2.12E-05 | 0.6409 | 0.0543967 | 0.0565408 | 0.33601 | 0.639298 |
| rs2354982 | T | C | 0.0823 | 0.0193 | 2.01E-05 | 0.8988 | 0.00387623 | 0.0958089 | 0.967728 | 0.9100245 |
| rs2744680 | A | G | -0.0667 | 0.0149 | 7.59E-06 | 0.8343 | 0.0506541 | 0.0744542 | 0.49629 | 0.847761 |
| rs4653570 | A | T | 0.0741 | 0.0148 | 5.54E-07 | 0.8092 | 0.0281285 | 0.0712444 | 0.692978 | 0.819248 |
| rs4669231 | A | G | 0.136 | 0.033 | 3.77E-05 | 0.0414 | 0.140775 | 0.13537 | 0.298373 | 0.0399198 |

Abbreviation: SNP, single nucleotide polymorphism; EA, Effect allele; OA, other allele; beta, estimate coefficient; se, standard error of coefficient estimate; eaf, effect allele frequency; ALS, amyotrophic lateral sclerosis.

**Table S17. Detailed information on the instrumental variables (IVs) for ALS and HHV-7 IgG in the reverse MR analysis.**

| **SNP** | **EA** | **OA** | **Exposure (ALS)** | | | | **Outcome (HHV-7 IgG)** | | | |
| --- | --- | --- | --- | --- | --- | --- | --- | --- | --- | --- |
|  |  |  | **beta** | **se** | **P** | **eaf** | **beta** | **se** | **P** | **eaf** |
| rs111629730 | T | C | 0.1987 | 0.0475 | 2.87E-05 | 0.0153 | -0.470122 | 0.300706 | 0.11796 | 0.0136584 |
| rs1214598 | A | G | -0.0555 | 0.0117 | 2.10E-06 | 0.371 | 0.0750146 | 0.0719625 | 0.297221 | 0.380301 |
| rs139248895 | T | G | -0.1898 | 0.0463 | 4.14E-05 | 0.0162 | 0.117916 | 0.24641 | 0.632268 | 0.0200596 |
| rs17524886 | T | C | -0.0491 | 0.011 | 8.06E-06 | 0.5154 | 0.0827529 | 0.0696186 | 0.234573 | 0.516714 |
| rs2123331 | A | G | -0.0489 | 0.0115 | 2.12E-05 | 0.6409 | -0.0660001 | 0.0734749 | 0.369043 | 0.639298 |
| rs2354982 | T | C | 0.0823 | 0.0193 | 2.01E-05 | 0.8988 | -0.156802 | 0.124694 | 0.208574 | 0.9100245 |
| rs2744680 | A | G | -0.0667 | 0.0149 | 7.59E-06 | 0.8343 | -0.135943 | 0.0970194 | 0.161155 | 0.847761 |
| rs4653570 | A | T | 0.0741 | 0.0148 | 5.54E-07 | 0.8092 | -0.0966367 | 0.0928697 | 0.298078 | 0.819248 |

Abbreviation: SNP, single nucleotide polymorphism; EA, Effect allele; OA, other allele; beta, estimate coefficient; se, standard error of coefficient estimate; eaf, effect allele frequency; ALS, amyotrophic lateral sclerosis.
